# A network approach to emotion regulation and symptom activation in depression and anxiety

**DOI:** 10.3389/fpubh.2024.1362148

**Published:** 2024-09-10

**Authors:** Ana Rita Rodrigues, Daniel Castro, Joana Cardoso, Filipa Ferreira, Carla Serrão, Carlos M. Coelho, Liliana Meira, Tiago B. Ferreira

**Affiliations:** ^1^University of Maia, Maia, Portugal; ^2^Center for Psychology, Faculty of Psychology and Education Science, University of Porto, Porto, Portugal; ^3^Instituto Politécnico do Porto, Escola Superior de Educação, Porto, Portugal; ^4^Centro de Investigação e Inovação em Educação (inED), Porto, Portugal; ^5^University Center for Research in Psychology (CUIP), University of the Azores, Ponta Delgada, Portugal

**Keywords:** emotion regulation strategies, depression, anxiety, network analysis, regulation of emotion systems survey

## Abstract

**Background:**

Emotions can be regulated through several regulatory strategies that are involved in the development of psychopathological symptoms. Despite the well-established association between psychopathology and emotion dysregulation, little is known about the relationship between individual symptoms of depression and anxiety and emotion regulation strategies (ERS), as well as between ERS themselves.

**Method:**

We conducted a cross-sectional study and examined the interactions between six ERS (reappraisal, engagement, rumination, suppression, arousal control, and distraction) and assessed their distinctive association with the activation of specific symptoms of depression and anxiety in a community sample of 376 adults (80.4% female; *M_age_* = 32.70; *SD_age_* = 11.80). The Regulation Emotion Systems Survey (RESS) was used to measure ERS. The Patient Health Questionnaire (PHQ-9) and the Generalized Anxiety Disorder (GAD-7) were used to assess psychological symptoms. An exploratory graph analysis was performed to examine the structural properties of the network of interactions between these behaviors. Additionally, to test the association of ERS with the activation of the depression symptoms network, an expected symptoms activity (ESA) was conducted.

**Results:**

Six communities were found that correspond to the six ERS. Rumination and suppression have a significant association with symptom activation (particularly low self-esteem), whereas reappraisal reduces symptomatic activation. The effect of arousal control, engagement, and distraction appears to depend on the remaining ERS rather than having much influence on their own.

**Conclusion:**

This study provides insight into how ERS interact with each other and with individual symptoms of depression and anxiety. Understanding the effects of these interactions on symptom activation and comorbidity can improve our understanding of psychopathology.

## Introduction

Regulation of our emotions is vital for both physical and mental health (1, 2). Emotion regulation has been defined as the ability to manage emotional responses to meet environmental demands and achieve one’s goals (3, 4), and is the result of cognitive, behavioral and physiological processes that influence the emotional experience and its expression (5–7). These components of the emotional experience structure the six strategies of emotion regulation that are typically used in daily life: cognitive reappraisal, expressive suppression, rumination, distraction, engagement, and arousal control. Distraction, rumination, and reappraisal are closely linked to the cognitive component of emotional experience; conversely, emotional suppression and engagement are related to the behavioral component, while arousal control is associated with the physiological component of emotional experience (8).

Several of these emotion regulation strategies have been implicated in the onset and maintenance of multiple psychiatric conditions (9, 10), especially depression (11, 12), and anxiety (11), while others have been associated with adaptive behaviors. For example, repeated use of rumination is associated with depression and anxiety symptoms (13), specifically guilt feelings, changes in appetite, agitation, sadness, fear of losing control, fear of the worst happening, nervous feelings, and inability to relax (14), while reappraisal is negatively associated with psychopathological symptoms (1, 2), specifically with pessimism and fear of the worst happening (14). Cognitive reappraisal has also been associated with other adaptive outcomes, such as lower levels of negative affect (15) and more effective interpersonal functioning (16). On the other hand, suppression has been associated with depressive symptoms, less life satisfaction, and less well-being (2). In the same way, some evidence suggests that the effectiveness of each emotion regulation strategy can vary depending on the situational context (17, 18). For example, Sheppes et al. (17) concluded that in the context of a low-intensity emotional situation, people tend to use reappraisal over distraction, while in the context of a high-intensity emotional situation, distraction was preferred. In this context, distraction may be inappropriate when long-term adjustment is needed (19) and reappraisal is ineffective in managing high-intensity emotional situations (20). These findings suggest that each of these strategies could be more adaptive and linked to positive outcomes or maladaptive and paired with negative outcomes when used in excess, exclusively, or depending on the context.

However, research on the psychopathological effect of ER strategies remains with some limitations that emerge from the relationship between ER strategies and psychopathology being studied by relating each of the strategies in isolation to a global estimate of the severity of symptoms associated with each mental disorder, ignoring the clinical heterogeneity and differential relations among their symptoms (14). First, the effect of the interaction between the different strategies remains unknown (1, 12, 21), which creates difficulties in discriminating the specific effects of each one of them. For example, rumination is implicated in different mental disorders, including depression (1) and anxiety (22), being associated with higher levels of negative affect in clinical and non-clinical samples (23). Second, the association of ER strategies with sum scores of psychopathology inventories appears to be unwarranted and inappropriate in the context of evidence, suggesting that psychopathological symptoms are etiopathogenically distinct (24) and affect psychosocial functioning differently as well (25). Associating each of the strategies with a psychopathology sum score is therefore a crude way of studying their effect and impairs our ability to pinpoint which specific symptoms each of these strategies tends to activate or deactivate. Consequently, this limits our understanding of the differential effect of ER strategies on specific symptoms and their adaptive or maladaptive function.

To surpass these limitations, network models of both psychopathology (26) and emotions (27, 28) have been proposed. In these network models, the components of psychopathology and emotions are conceptualized as a result of the interactions of their components. This conceptualization provided important information on the structure of psychopathology (29) and the overlap of emotional components between emotions (28). For example, identify symptoms that belong to more than one mental disorder (30) or emotional components that are present in more than one emotion (28). However, limiting the network models to only one type of element (e.g., symptoms or emotion components) does not allow for the identification of the differential effect that might occur between related elements of different natures. Due to this, in psychopathological networks, elements other than symptoms are included in psychopathological symptoms networks to assess their differential effect on symptoms, for example, genetic elements (31), cognitive elements (32), and environmental elements (33). This is important since the identification of the differential effect of a specific element could provide significant information to improve the personalization and efficacy of treatment by identifying productive treatment goals (26, 34). Network analysis, which focuses on the practical application of techniques such as multivariate regression equations with regularization, is commonly used in these models (35).

The ER strategies are one of the most important emotion components in the psychotherapeutic context and the focus of many psychotherapeutic interventions (36–39), and have been suggested to be important treatment targets (40). An initial exploration of their differential effect on specific elements of mental disorders through network analysis has begun to appear in the literature (14). These studies corroborated the theoretical proposals of the emotion and psychopathology network models, by identifying interactions between the ER strategies (28, 41), and the differential effect of these strategies on specific symptoms (14). However, previous studies focused primarily on one mental disorder (41, 42), leaving unanswered questions about the differential effect of ER strategies across mental disorders (1), which are essential for the clarification of comorbidity structures.

Furthermore, simply identifying these connections between ER strategies and specific symptoms does not provide enough information on the adaptive or maladaptive role of these strategies. This must be combined with an understanding of the effect of each ER strategy in exacerbating or improving each of the symptoms. Together, this knowledge is crucial to supporting the needs of psychotherapists in routine practice, such as identifying treatment goals and the selection of therapeutic strategies. Lunansky et al. (43) have recently proposed a method, Expected Symptom Activity (ESA), to test the effect of external factors on the activation of symptoms networks. Through ESA changes in the overall network connectivity can be inferred by simulating changes in specific nodes. The application of ESA to ER strategies could provide important information on the role of these strategies in activating and deactivating the connectivity of symptoms networks, which, according to the psychopathology network theory (44), is a marker of psychopathology and its emergence (45), and can inform us about the adaptive or maladaptive role of ER strategies.

Despite the established link between psychopathology and emotion dysregulation, little is known about the relationship between the specific symptoms of depression and anxiety and the different strategies of emotion regulation. A testable detailed model of these relationships is required that does not merely focus on sum-scores and assumes that ER strategies are not used in isolation, but in combination. The importance of testing this model is related to the fact that it is crucial to understand how ER strategies interact with each other, as well as with specific symptoms. Understanding this will allow us to understand the effect these interactions between ER strategies have on symptom activation. Likewise, exploring these associations at a more detailed level also helps us to understand the comorbidity or co-occurrence between these mental disorders. In this context, the network approach could provide important insights on both the adaptive and maladaptive role of ER strategies in the development and maintenance of psychopathological disorders and the differential implication of different ER strategies in the comorbidity structures of mental disorders. Consequently, our study aims (1) to map the interactions between ER strategies, depression, and anxiety; (2) to test the differential effect of each ER strategy.

## Materials and methods

### Participants

A community-based sample of 376 participants (80.4% female; *M_age_* = 32.70; *SD_age_* = 11.80) was admitted to the present study. The eligibility criteria for participation were: (1) age over 18 years and (2) Portuguese-language proficiency. Participants’ sociodemographic characteristics are summarized in Table 1. In this online cross-sectional survey, emotional regulation strategies (assessed by RESS), as well as depressive and anxiety symptoms (measured by PHQ-9 and GAD-7, respectively) were collected concurrently. There are no missing data.

**Table 1:** Sociodemographic characteristics of the sample.

Variable	Total (*N* = 376)
Age in years, *M* (*SD*)	32.70 (11.80)
Gender	
Male, *n* (*%*)	66 (17.6)
Female, *n* (*%*)	310 (80.4)
Marital status	
Single, *n* (*%*)	233 (62)
Married, *n* (*%*)	113 (30.1)
Divorced, *n* (*%*)	28 (7.4)
Widowed, *n* (*%*)	2 (0.5)
Education level	
Primary, *n* (*%*)	12 (3.2)
Secondary, *n* (*%*)	94 (25)
Higher education or graduate, *n* (*%*)	270 (71.8)
Occupation	
Employee, *n* (*%*)	191 (50.8)
Student, *n* (*%*)	99 (26.3)
Worker/Student, *n* (*%*)	59 (15.7)
Unemployed, *n* (*%*)	27 (7.2)
Psychological, psychiatric and/or pharmacological treatment	
Yes, *n* (*%*)	52 (13.8)
No, *n* (*%*)	324 (86.2)

### Measures

#### Regulation of emotion systems survey (RESS)

RESS (8) is a 38-item self-report questionnaire designed to assess an individual’s propensity to use six emotion regulation strategies to decrease their experience of negative emotions: (a) distraction, (b) rumination, (c) reappraisal, (d) suppression, (e) engagement, and (f) arousal control. Suppression involves actively concealing the outward expression of an emotional experience (e.g., “Hiding my feelings”). Engagement entails actively participating in an emotion, intensifying the expressive aspects to moderate the emotional experience (e.g., “Expressing my feelings”). Reappraisal is related to altering an emotional experience, changing the way one thinks about it (e.g., “Looking at the situation from several different angles”). Arousal control refers to managing the physiological arousal linked to emotions (e.g., Trying to slow my heart rate and breathing”). Distraction entails diverting attention away from the emotional experience (e.g., “Immediately working on something to keep myself busy”). Lastly, rumination involves persistently dwelling on an emotional experience and its implications (e.g., Thinking again and again about what went wrong”). Participants rate each item on a 5-point Likert scale ranging from 1 (never) to 5 (always). The present scale revealed good psychometric properties, with the subscales presenting Cronbach’s alpha values ranging from 0.88 to 0.94 (8).

#### Generalized anxiety disorder (GAD)

GAD-7 (46, 47) is a 7-item self-report scale that measures the severity of symptoms of generalized anxiety disorder in the last 2 weeks. Participants rate each item on a scale from 0 (never) to 3 (almost every day). The internal consistency of the original scale was very satisfactory (Cronbach *α* = 0.92) and test–retest reliability was also good [intraclass correlation = 0.83; (46)]. The adaptation of this instrument for the Portuguese population also revealed satisfactory results regarding internal consistency (Cronbach’s alpha = 0.88), with all items showing high test–retest correlation coefficients (47).

#### Patient health questionnaire-9 (PHQ-9)

The PHQ-9 (48); Portuguese version (49) is a 9-item self-report scale that assesses symptoms of depression. The nine items are rated on a 4-point Likert scale: 0 (never) to 3 (almost every day). The internal reliability of the original scale was very satisfactory, with a Cronbach’s α of 0.89 (48) and the Portuguese version also showed satisfactory results in terms of internal consistency (Cronbach’s alpha = 0.86; (49)).

### Procedure

The present study was carried out in Portugal. Since there was no Portuguese version of the RESS available, we started with the adaptation of the RESS for the Portuguese language and population. After obtaining authorization from the original authors, experienced researchers and bilingual experts performed a method of multiple translation and reconciliation associated with back-translation to confirm the fidelity of the Portuguese version (50). To assess the quality of the translation, a sample of 64 participants outside the study initially answered the questionnaire. Participants did not report any problems with item clarity or item bias (i.e., gender, ethnicity, socioeconomic, or cultural status).

After cultural adaptation of RESS, the current study was spread through mailing lists, personal contacts, and social media accounts. Data collection was carried out through an online survey between May and September 2020. The study was approved by the Ethics Committee of the Faculty of Psychology and Educational Sciences of the University of Porto (Ref. 2020/03-4b) and all participants gave their informed consent before admission to the study.

### Data analysis

#### Exploratory graph analysis

Data analysis was performed in R [version 1.4; (51)]. Exploratory graph analysis [EGA; (52, 53)], implemented in the R package EGA (54), was used to estimate the network of connections between the items of the RESS, as well as to determine the number and composition of the communities that make up this network. Based on the raw scores assigned by participants to each of the items on the scale, EGA uses a Gaussian graphic model (GGM) to estimate the strength of pairwise interactions between these items (which can be interpreted as partial correlation coefficients). To select the most parsimonious network while controlling for type I errors, the GGM is estimated through the least absolute shrinkage and selection operator (LASSO) (55), and the extended Bayesian information criterion (56) to control the network sparsity. After estimating the network of connections between items, the most closely linked subgroups of items were identified through the *walktrap* algorithm (57). These subgroups are called communities, which correspond to the factors in the traditional exploratory factor analysis and, in the case of the RESS, to the emotion regulation strategies. To measure the centrality of each item, strength was used. Strength corresponds to the sum of the absolute weights of the nodes’ connections (35, 58). To measure bridge symptoms, the Networktools R package [version 1.2.3; (59)] was used. Bridge symptoms are symptoms that connect distinct clusters of symptoms related to different mental disorders or subgroups of symptoms within the same mental condition (60). The R package *qgraph* (61) was used for network visualization. See the Supplementary material for the results of the EGA of the RESS. Supplementary Figure S1 displays the network of connections between the 38 items that constitute the RESS. The Portuguese version of RESS reproduces the original version (8).

### Expected symptom activity

Then an expected symptom activity (ESA) was performed, as proposed by Lunansky et al. (43), to investigate how the activity of the symptoms is altered in the presence or absence of specific ER strategies. First, the network of interactions between emotion regulation strategies and symptoms was estimated with a mixed graphical model (MGM), using the *bootnet* package in R with *mgm* default, using 10-fold cross-validation to select the regularization parameter (35). MGM was selected since it can account for the different measurement scales in the data, ordinal (PHQ-9 and GAD-7 items, corresponding to depression and anxiety symptoms) and continuous (RESS ER strategies). The potential confounding effect of sociodemographic variables was tested as in previous studies (62) and is presented in Supplementary material.

Second, baseline symptom activity (BSA) was estimated. This was carried out by calculating the mean of all network symptoms (PHQ-9 and GAD-7). Then, to study how ER strategies affect symptom activity, ER strategies were conditioned on all possible values, where the highest values indicate a higher frequency of use. Since the range of each ER strategy varies, the conditioning of each strategy was performed accordingly. The suppression, engagement and reappraisal strategies were conditioned between 8 and 40, arousal control and distraction were conditioned with values between 4 and 20, and rumination was conditioned with values between 6 and 30. After conditioning each ER strategy, ESA was obtained by summing the resulting symptoms’ means.

Furthermore, as previous studies have suggested that interactions between ER strategies influence how they affect symptom activation (14), we simulated three different scenarios of ER strategies to explore changes arising from the use of a specific emotional regulation strategy in different emotional regulation scenarios. In the first scenario, we conditioned each ER strategy in all possible values while keeping the remaining ER strategies to their minimum possible score. For example, while conditioning reappraisal to all its possible values, we kept the remaining strategies with their minimum possible value (i.e., Engagement, Suppression, and Reappraisal = 8; Rumination = 6; Arousal control and Distraction = 4). In the second scenario, the conditioning of each ER strategy was performed while the remaining ER strategies were kept at the median value of the scale (i.e., Engagement, Suppression, and Reappraisal = 24; Rumination = 18; Arousal control and Distraction = 12). For the last scenario, conditioning was performed with the remaining ER strategies kept at the maximum possible value (i.e., Engagement, Suppression, and Reappraisal = 40; Rumination = 30; Arousal control and distraction = 20).

## Results

### Mixed graphical model including ER strategies, depressive, and anxiety symptoms

**Figure 1:**
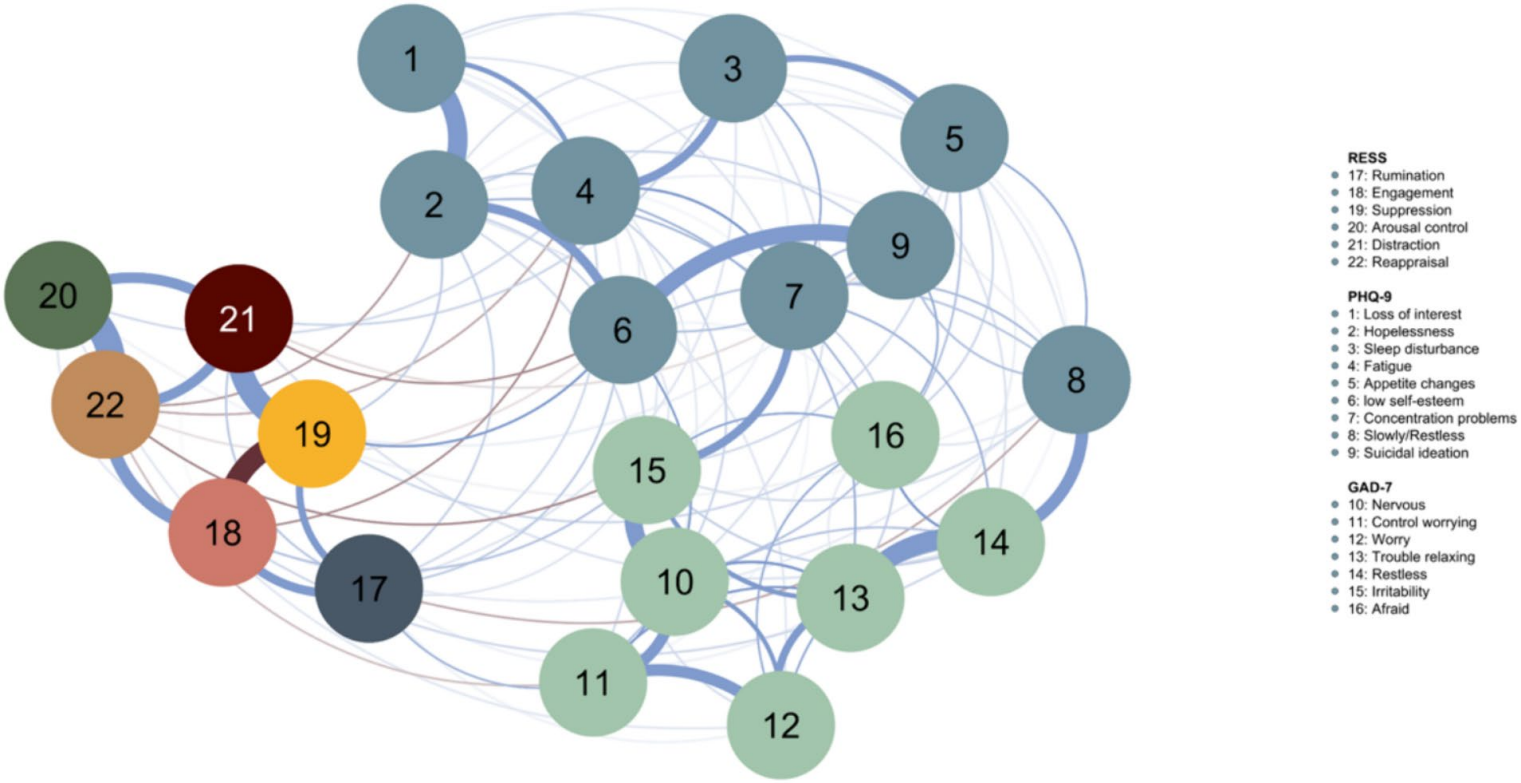
Mixed graphical model including ER strategies and depressive and anxiety symptoms. The blue nodes represent depressive symptoms (PHQ-9). Green nodes represent anxiety symptoms (GAD-7). Blue lines indicate positive associations, and brown lines represent negative associations. The width of the edges and the intensity of the color represent the strength of the edge.

**Figure 2:**
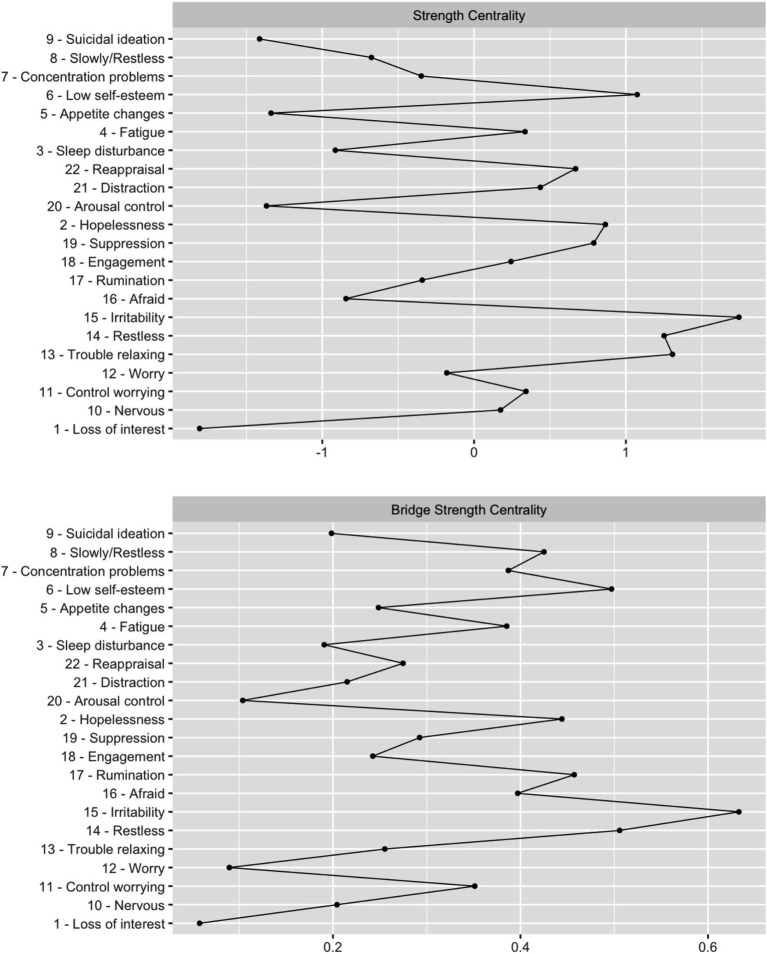
Centrality plot for strength and bridge strength. Centrality plot for the strength and bridge strength of each node (depressive and anxiety symptoms, and ER strategies).

Figure 1 presents the network of interactions between depression, anxiety symptoms, and ER strategies (network density = 0.485). This network comprises 131 connections, 10 of which are negative. The properties of the network are shown in Table 2. Figure 2 presents the centrality plot of strength and bridge strength. Globally, the strongest vertices are irritability (15), restlessness (14), low self-esteem (6), and trouble relaxing (13); the least strong are arousal control (20) and appetite changes (5). The strongest ER strategies in the network are suppression (19) and reappraisal (22), and the least strong is arousal control (20). In terms of symptoms, those with less strength are suicidal ideation (9) and loss of interest (1). Of the strongest symptoms, only irritability and low self-esteem are directly related to ER strategies. Bridge symptoms with the highest strength are irritability and low self-esteem. The network showed adequate stability with a CS-coefficient of 0.285 for node strength and a CS-coefficient of 0.596 for the edges. Stability plots for this network can be found in Supplementary Figures S3–S5.

**Table 2:** Global proprieties of the network, including ER strategies, depressive and anxiety symptoms.

Network propriety	Scores
Average path length	1.519
Transitivity	0.512
Clustering coefficient	0.520

Global proprieties of the network. The average path length corresponds to the shortest path between all pairs of nodes. Transitivity is the ratio of triangles and connected triples in the graph. The clustering coefficient quantifies the degree to which nodes in a graph tend to cluster together.

### Expected symptom activity with the RESS

The ER strategies were positively related to each other, in general: rumination was positively associated with suppression, engagement, and distraction; engagement was positively related to suppression, arousal control, distraction, and reappraisal; suppression was positively associated with distraction and reappraisal; and distraction was positively associated with reappraisal. Engagement and distraction had more links to other ER strategies than to specific symptoms. Arousal control displayed an equal number of links to symptoms and ER strategies. The remaining strategies (rumination, suppression, and reappraisal) displayed more links to symptoms than other ER strategies.

ER strategies were also positively related to depression and anxiety symptoms, such as rumination with uncontrollable worry, suppression with low self-esteem, and control of arousal with fatigue, restlessness, and worry. Furthermore, negative associations were observed between various ER strategies and depression and anxiety. For example, reappraisal was negatively associated with hopelessness, fatigue, low self-esteem, uncontrollable worry, and irritability. Rumination, engagement, and distraction only showed negative links to depressive symptoms. Specifically, engagement was negatively associated with symptom fatigue, distraction was negatively linked with low self-esteem and suicidal ideation, and rumination was linked with restlessness. The arousal control was the only one to show only positive connections in the network, both with other ER strategies and with symptoms of depression and anxiety.

**Figure 3:**
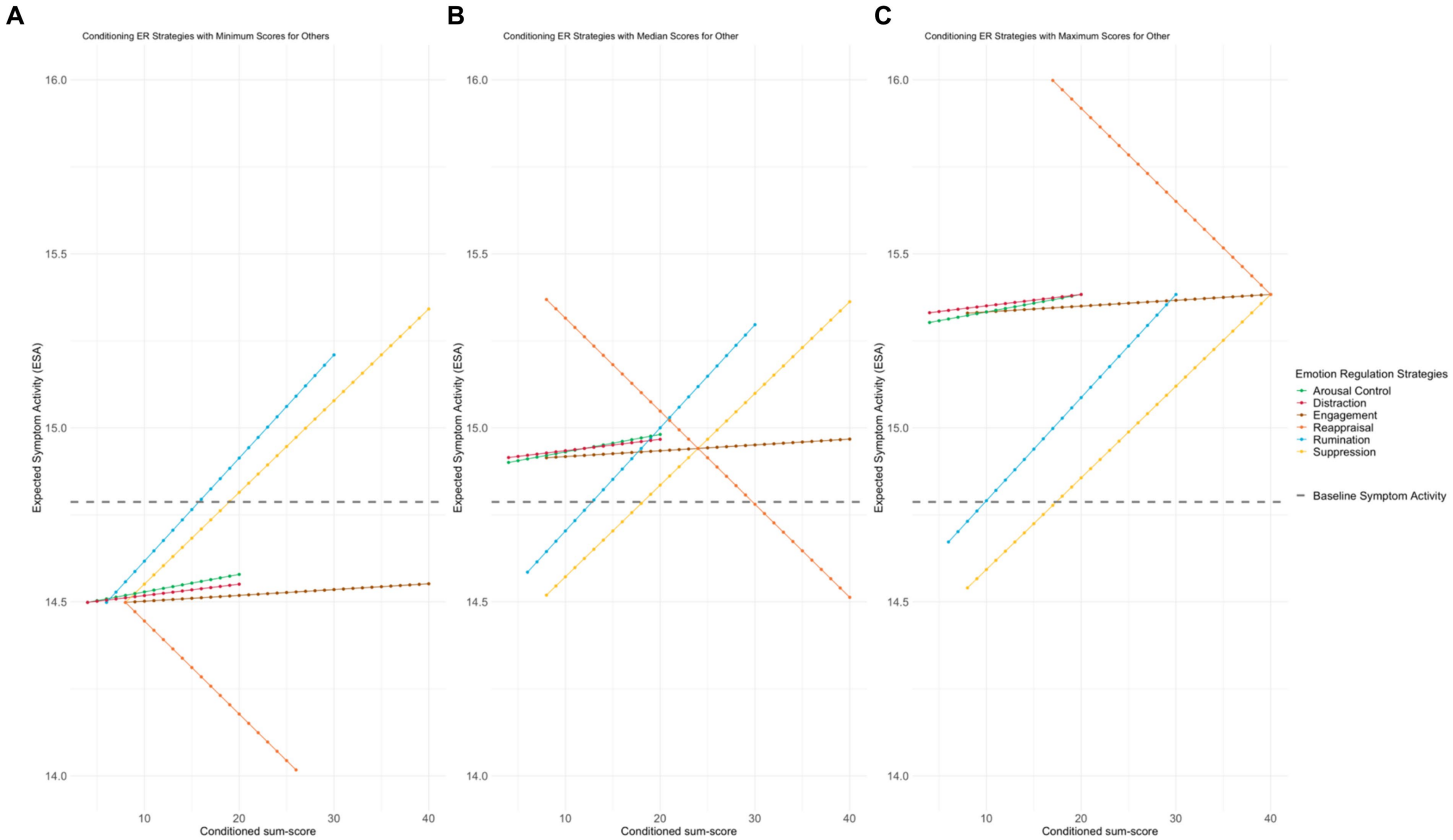
Simulations conducted on ESA. Figure illustrates the simulations conducted on ESA within each of the three simulated scenarios. Each panel depicts the alterations observed in the network when a specific strategy is simulated on all of its values, while the remaining strategies are held constant at the corresponding scenario values (minimum, median and maximum). The *x*-axis represents the sum scores of each RESS subscale (emotional regulation strategy), while the *y*-axis corresponds to the ESA values. ER, emotional regulation; ESA, expected symptom activity; **(A)** represents ER ESA with minimum level in the remaining strategies; **(B)** represents ER ESA with median level in the remaining strategies; **(C)** represents ER ESA with maximum level in the remaining strategies. The gray line corresponds to the ESA baseline. The yellow line represents suppression, the blue line represents rumination, the orange line represents reappraisal, the brown line represents engagement, the red line represents distraction, and the green line represents arousal control.

Figure 3 shows each ESA of the ER strategy with minimum, median and maximum levels in the remaining strategies. The ESA of the baseline model was 14.787 (see Supplementary Table S1 for detailed information). In the first scenario (Figure 3A), when we conditioned each of the ER strategies to the minimum sum-score of the remaining ER strategies, rumination and suppression increased the symptom activation, especially from sum-scores 16 and 19 forward, respectively. Regarding rumination, the minimum activation in the symptoms network was 14.498 (the difference from baseline ESA was 0.289) and the maximum was 15.209 (the difference from baseline ESA was −0.422), suggesting that rumination increases the activation of symptoms. In the case of suppression, the minimum activation of symptoms was 14.499 and the maximum was 15.342 (the difference from the baseline ESA model was 0.289 and −0.554, respectively), also suggesting that it generates a greater activation of symptoms. Regarding reappraisal, it appeared to minimize the activation of symptoms: the minimum symptom activation of reappraisal was 14.499 and the maximum was 13.643 (evidencing a difference of 0.289 and 1.144, respectively, compared to the baseline ESA model). In this scenario, engagement, arousal control, and distraction do not seem to be associated with changes in symptoms’ activation.

In the second scenario (Figure 3B), when all ER strategies were in the median except the one being tested, rumination appeared to need a lower sum-score value (13 points), compared to the previous scenario, to begin generating greater activation of symptoms than exists at baseline (14.793 evidencing a difference of −0.006 with baseline ESA). Furthermore, when all ER strategies were in the median and there were minimal reappraisal values, this alone generated an increase in symptom activation. For instance, when we have very low scores, such as 8 points, which is the minimum sum-score for reappraisal, the ESA increases to 15.369 (showing a difference of −0.582 compared to the ESA baseline model). In this sense, the individual must have at least 30 points in the sum score of the subscale so that there is no greater activation of symptoms than what initially appears in the baseline ESA model. In addition, when all ER strategies were in the median, engagement, arousal control, and distraction resulted in higher symptom activation in all conditioned sum-scores. Specifically, ESA of engagement varies between 14.914 to 14.968 (the difference compared to the base ESA model that varies between −0.127 and −0.180), arousal control from 14.901 to 14.981 (the difference compared to the base ESA model that varies between −0.113 and −0.194), and distraction from 14.915 to 14.968 (the difference compared to the base ESA model that varies between −0.128 and −0.180).

In the third and last scenario (Figure 3C), when all ER strategies were at their maximum, reappraisal failed to generate an activation of symptoms lower than the baseline ESA model (ESA values decrease from 16.239 to 15.383 compared to the baseline model). On the other hand, when suppression showed minimum values while the others showed maximum values, it initially did not generate a higher activation of symptoms than found in the baseline ESA model. In this case, to get more activation of symptoms than what is initially displayed by the baseline ESA model, the sum-score must be at least 18 points. In the case of rumination, this strategy seems to need a much lower sum-score (10 points) compared to the first and second scenarios (16 and 13 points, respectively) to generate a symptom activation higher than that found in the baseline ESA model. Engagement, arousal control, and distraction, when all other strategies were conditioned to their maximum values, generated greater symptomatic activation for any of the conditioned sum-scores.

## Discussion

Psychological research has changed its focus to interactions between symptoms (44) and to the differential association of psychological mechanisms with specific symptoms (63). ER strategies are among the most studied mechanisms of mental disorders (9, 10); however, research focusing on the differential role of these strategies is still lacking (14). To surpass this, we constructed a network model of interactions between ER strategies, depression, and anxiety symptoms and explored the combined effects of different ER strategies on the activation and deactivation of these symptoms.

Suppression and reappraisal are the ER strategies with the strongest connections in the network. As an antecedent-focused ER strategy, reappraisal generally manifests itself before emotional experiences are fully produced (64). Therefore, this involves the reinterpretation of the meaning of an emotional situation to change its emotional impact (65). In contrast, suppression occurs after emotional experiences are fully generated and involves inhibiting outward emotional expression (5, 65). The literature (66, 67) has argued that in terms of mental health gains, more frequent use of suppression and less frequent use of reappraisal are associated with depression and anxiety, as reappraisal has been suggested as an ER strategy capable of decreasing negative affect, and suppression, on the other hand, has been associated with physiological arousal.

In the current study, reappraisal was the only ER strategy capable of reducing the activation of symptoms, therefore, acting as a protective factor against their escalation and maintenance. However, the results suggested that the protection offered by reappraisal is bounded by the levels at which the remaining ER strategies are used by individuals. If the remaining ER strategies increase, reappraisal fails in its ability to reduce the activity of symptoms. The combined results are shown in Figures 3B,C. When individuals use a lot of dysfunctional ER strategies (rumination, suppression) there is a moment when the negative consequences of this overuse exceed the ability of reappraisal to stabilize the entire system and compensate for this negative effect. These findings go beyond the conclusions obtained by Aldao et al. (1), which suggest that the adoption of reappraisal as an emotional response mechanism is associated with the reduction of negative emotions and physiological arousal *per se* and, therefore, naturally considered as a protective factor against depression and anxiety.

Similarly, the effect of rumination on the activation of symptoms also seems to be influenced by the behavior of other ER strategies. For example, in the scenario with maximum ER strategies, rumination requires a lower sum-score to cause a high level of symptom activation. This seems to be consistent with what D’Avanzato et al. (68) proposed, as they argue that greater use of maladaptive strategies (e.g., suppression, rumination) and less use of adaptive strategies (specifically, reappraisal) it is a general feature of psychopathology (particularly regarding depression and anxiety).

In contrast, the results suggest that suppression does not seem to depend on the other strategies. This is because regardless of whether the remaining ER strategies are at minimum or maximum levels, suppression always has the effect of increasing symptomatic activation (in particular, from sums greater than 18 in Figures 3A,B, and 19 in Figure 3C). These findings seem to overcome some limitations found in recent theoretical (69) and empirical (3) literature, insofar as a contextual approach to ER is advocated. Thus, the ability of some ER strategies to function as protective or risk factors depends not only on the context but also on the levels at which they are used (under or overuse), as well as on the behavior of the remaining ER strategies, acting as a single and a global system.

Therefore, suppression and rumination seem to work as risk factors in the maintenance and/or activation of symptoms. This appears to be in line with recent research (70), which has consistently confirmed significant links between suppression, rumination, depression, and anxiety. Furthermore, the results of our study seem to agree with what was proposed by Aldao et al. (1) in a meta-analytic review of cognitive ER strategies, as they suggest that the presence of maladaptive ER strategies is more harmful than the absence of positive strategies. This can be seen in our study; when maladaptive ER strategies are present, such as rumination or suppression, adaptive ER strategies (reappraisal) appear not to be sufficiently capable of reducing symptoms.

The other ER strategies, specifically engagement, arousal control, and distraction, seem to depend on the activity of other ER strategies rather than having much influence on their own. Thus, when the other ER strategies have low levels, for example, engagement, arousal control, and distraction do not seem to increase symptom activation. However, in both the second and third scenarios, they result in greater symptomatic activation for any of the conditioned sum-scores. This appears to be consistent with previous evidence suggesting that the effect of each ER strategy varies by situational context (17, 18, 71). An explanation for this could be the fact that they have a greater number of links with other ER strategies compared to links with specific symptoms. On the other hand, as shown in the first scenario, they do not appear to be associated with changes in the symptom activation network. If these strategies are related to psychopathology, that is, with the absence of mental disorders such as depression, they are not associated with symptomatic activation, but perhaps with other processes, such as cognitive processes (72) or executive functions.

The present study suggests that, depending on the level of activation that is simulated, ER strategies will have a differential effect on activation of symptoms. Furthermore, changes on symptomatic activation depend not only on the strategy that is being conditioned or manipulated, but on the level (sum-score) of activation of all the others. Therefore, symptomatic activation is not only associated with a particular ER strategy but also depends on all others. These results seem to meet what is defended by network theory (73), since it is understood that the elements (in this specific case, the emotional regulation strategies) of a complex network interact and influence each other. In addition, the results appear to indicate that the reduced use of all ER strategies leads to less than baseline symptomatic activation compared to the medium or higher use of all ER strategies.

This study also presents several limitations. First, most of the participants were female and have a higher education level, which limits generalizability. Second, our study had a community sample, which excluded comparisons of individuals with and without mental disorders, namely depression and anxiety. In this sense, additional studies should be conducted across different populations and by other research groups (i.e., with diagnosis of anxiety and depression diagnosis) to investigate whether the present findings replicate. Third, another limitation of our study is the presence of additional components beyond the GAD7, PHQ9, and RESS strategies, such as age and sex, which were not incorporated into the network. These factors could also potentially affect ER strategies and symptoms of depression and anxiety. Fourthly, we highlight that the absence of directionality in our network is a significant limitation for assessing causality. Although Expected Symptom Activity (ESA) informs us about the effect of specific changes occurring in the network, this limitation remains substantial. Furthermore, the study of causality through cross-sectional data is a growing field of research in psychology and other areas through network analysis. This approach presents challenges in interpreting dynamic interactions between symptoms and emotion regulation strategies that must be addressed in future studies. Lastly, this is a cross-sectional study. This naturally excludes estimates of important features of the network, such as the direction of the edges or cyclic self-reinforcing edges. For example, it does not allow for causal inference. In this way, follow-up studies may be interesting in determining causality, through temporal time-series studies. According to Fried and Cramer (74), the temporal nature of psychopathological symptoms and emotions remains a problem. For example, more studies should consider some questions such as do symptoms or emotions/ER strategies evolve over a time span of minutes, hours, or days? Is this time period different for specific variables or associations? Therefore, we believe that it will be useful to replicate our findings with additional methodologies, such as ecological momentary assessment [EMA; (23)]. This replication will clarify which specific emotional regulation strategies need to be developed in the patient to carry out interventions that are increasingly individualized, effective, and adjusted to the needs of different individuals in a psychotherapeutic context, considering the characteristics of the patients (for example, baseline symptomatology) and their ability to respond to emotion regulation. Despite the limitations noted, this study provides valuable information for future research, advances in generalizability, and a clearer understanding of how emotion regulation (ER) strategies can influence the severity of symptoms and the optimal use of these strategies. By examining the relationship between different ER strategies and depression and anxiety symptoms, the study highlights the potential pathways through which these strategies can exacerbate or alleviate symptoms. This information is crucial for designing more targeted and effective interventions. Furthermore, the findings offer a framework for exploring the complex interactions between various ER strategies and specific symptoms, paving the way for personalized treatment approaches that consider individual differences in ER patterns and their effects on mental health outcomes.

## Data Availability

The raw data supporting the conclusions of this article will be made available by the authors, without undue reservation. This study’s supporting data are available from the corresponding author, upon request.
