# Supplementary material for: A network approach to emotion regulation and symptom activation in depression and anxiety
Source: Front Public Health. 2024 Sep 10;12:1362148. doi: 10.3389/fpubh.2024.1362148 (PMC11420018; doi:10.3389/fpubh.2024.1362148)
Supplement: Supplementary file 1 [file Data_Sheet_1.docx]

Supplementary Materials

**A Network Approach to Emotion Regulation and Symptom Activation in Depression and Anxiety**

**Index**

1. Exploratory Graph Analysis of RESS
2. ER strategies, conditioned sum-scores, ESA, and difference with baseline ESA
3. Graphical representation of the Exploratory Analysis of RESS
4. Strength centrality plot of RESS items
5. Stability Plots Mixed Graphical Model
6. Analysis of the confounding effects of sociodemographic variables

**1. Exploratory Graph Analysis of the RESS**

Regarding EGA, the Portuguese version of RESS was analyzed according to the model of complex networks and reproduces the original version (De France & Hollenstein, 2017).

The strongest connection (.69) was the edge between node 37 (“*trying to think of the emotional event in a more positive light*”) and node 38 (“*trying to see the situation in a more positive light*”), both belonging to *reappraisal* strategy. Other examples of strong connections found were edge between node 6 and node 8 pertaining to *arousal control* *(“focusing on slowing my heart rate and breathing”; “trying to slow my heart rate and breathing”)* and edge 31-32 belonging to *suppression (“making an effort to hide my feelings”; “concealing how I was feeling”).*

However, some items were negatively connected to each other and connected different domains of emotion regulation (ER). For example, edge 3-4 connected suppression and engagement *(“acting like I was not upset”; “letting my emotions show”)* and edge 14-25 connected reappraisal and suppression *(“trying to see the emotional event from a different perspective”; “hiding my feelings”)*.

Six communities were identified that corresponded to the 6 Emotion Regulation (ER) strategies that constitute the RESS and that mirror the original version (De France et al., 2017). The median was six dimensions. The structural consistency result showed that all dimensions were stable. All items from the total of six dimensions were at the range of 1, where they can be considered stable leading to a good structural consistency.

The items 14, 18, 20, 21, 24, 36, 37, and 38 covered community 1 (*reappraisal*). Community 2 *(engagement)* consisted in eight items: 4, 5, 7, 11, 16, 19, and 23. Community 3 *(rumination)* consisted in six items and included items 1, 10, 17, 28, 33, and 34. Community 4 *(suppression)* consisted in eight items and included items 3, 12, 15, 25, 26, 31, 32, and 35. Community 5 *(control arousal)* consisted in four items and included items 6, 8, 37, and 30. Finally, community 6 *(distraction)* covered four items: 9, 13, 22, and 29.

The nodes *“Concealing how I was feeling”* (RESS-32; suppression), *“Expressing my feelings”* (RESS-19; engagement), *“Thinking again and again about what went wrong”* (RESS-33; rumination), and *“Thinking about the emotional event again and again in my mind”* (RESS-34; rumination) were the nodes with strongest connections in the network. The nodes *“Thinking repeatedly about what was bothering me”* (RESS-1; rumination), *“Using facial expressions to show that I was upset”* (RESS-2; engagement), *“Acting like I was not upset”* (RESS-3; suppression), and *“Continually trying to decide what went wrong”* (RESS-28; rumination) were the nodes with the weaker connections in the network.

**2. ER strategies, conditioned sum-scores, ESA, and difference with baseline ESA**

*Table S1.*

*ER strategies, conditioned sum-scores, ESA, and difference with baseline ESA*

| **ER Strategy** | **Conditioned sum-score** | **ESA** | | | **Difference with baseline ESA model** | | |
| --- | --- | --- | --- | --- | --- | --- | --- |
|  | | **Min** | **Median** | **Max** | **Min** | **Median** | **Max** |
| Rumination | 6 | 14.49852 | 14.58537 | 14.67222 | .288713980 | .201866024 | .115018067 |
| Rumination | 7 | 14.52815 | 14.61500 | 14.70185 | .259083873 | .172235916 | .085387960 |
| Rumination | 8 | 14.55778 | 14.64463 | 14.73148 | .229453765 | .142605808 | .055757852 |
| Rumination | 9 | 14.58741 | 14.67426 | 14.76111 | .199823657 | .112975700 | .026127744 |
| Rumination | 10 | 14.61704 | 14.70389 | *14.79074 | .170193549 | .083345593 | *-.003502364 |
| Rumination | 11 | 14.64667 | 14.73352 | 14.82037 | .140563441 | .053715485 | -.033132472 |
| Rumination | 12 | 14.67630 | 14.76315 | 14.85000 | .110933333 | .024085377 | -.062762580 |
| Rumination | 13 | 14.70593 | *14.79278 | 14.87963 | .081303225 | *-.005544731 | -.092392688 |
| Rumination | 14 | 14.73556 | 14.82241 | 14.90926 | .051673118 | -.035174839 | -.122022795 |
| Rumination | 15 | 14.76519 | 14.85204 | 14.93889 | .022043010 | -.064804947 | -.151652903 |
| Rumination | 16 | *14.79482 | 14.88167 | 14.96852 | *-.007587098 | -.094435055 | -.181283011 |
| Rumination | 17 | 14.82445 | 14.91130 | 14.99815 | -.037217206 | -.124065162 | -.210913119 |
| Rumination | 18 | 14.85408 | 14.94093 | 15.02778 | -.066847314 | -.153695270 | -.240543227 |
| Rumination | 19 | 14.88371 | 14.97056 | 15.05741 | -.096477422 | -.183325378 | -.270173335 |
| Rumination | 20 | 14.91334 | 15.00019 | 15.08704 | -.126107529 | -.212955486 | -.299803442 |
| Rumination | 21 | 14.94297 | 15.02982 | 15.11667 | -.155737637 | -.242585594 | -.329433550 |
| Rumination | 22 | 14.97260 | 15.05945 | 15.14630 | -.185367745 | -.272215702 | -.359063658 |
| Rumination | 23 | 15.00223 | 15.08908 | 15.17593 | -.214997853 | -.301845810 | -.388693766 |
| Rumination | 24 | 15.03186 | 15.11871 | 15.20556 | -.244627961 | -.331475917 | -.418323874 |
| Rumination | 25 | 15.06149 | 15.14834 | 15.23519 | -.274258069 | -.361106025 | -.447953982 |
| Rumination | 26 | 15.09112 | 15.17797 | 15.26482 | -.303888177 | -.390736133 | -.477584090 |
| Rumination | 27 | 15.12075 | 15.20760 | 15.29445 | -.333518284 | -.420366241 | -.507214197 |
| Rumination | 28 | 15.15038 | 15.23723 | 15.32408 | -.363148392 | -.449996349 | -.536844305 |
| Rumination | 29 | 15.18001 | 15.26686 | 15.35371 | -.392778500 | -.479626457 | -.566474413 |
| Rumination | 30 | 15.20964 | 15.29649 | 15.38334 | -.422408608 | -.509256564 | -.596104521 |
| Engagement | 8 | 14.49852 | 14.91417 | 15.32981 | .288713980 | -.126932692 | -.542579365 |
| Engagement | 9 | 14.50019 | 14.91584 | 15.33149 | .287041319 | -.128605353 | -.544252026 |
| Engagement | 10 | 14.50187 | 14.91751 | 15.33316 | .285368658 | -.130278014 | -.545924687 |
| Engagement | 11 | 14.50354 | 14.91918 | 15.33483 | .283695997 | -.131950675 | -.547597348 |
| Engagement | 12 | 14.50521 | 14.92086 | 15.33650 | .282023336 | -.133623337 | -.549270009 |
| Engagement | 13 | 14.50688 | 14.92253 | 15.33818 | .280350675 | -.135295998 | -0.550942670 |
| Engagement | 14 | 14.50856 | 14.92420 | 15.33985 | .278678014 | -.136968659 | -.552615331 |
| Engagement | 15 | 14.51023 | 14.92588 | 15.34152 | .277005352 | -.138641320 | -.554287992 |
| Engagement | 16 | 14.51190 | 14.92755 | 15.34319 | .275332691 | -.140313981 | -.555960654 |
| Engagement | 17 | 14.51357 | 14.92922 | 15.34487 | .273660030 | -.141986642 | -.557633315 |
| Engagement | 18 | 14.51525 | 14.93089 | 15.34654 | .271987369 | -.143659303 | -.559305976 |
| Engagement | 19 | 14.51692 | 14.93257 | 15.34821 | .270314708 | -.145331965 | -.560978637 |
| Engagement | 20 | 14.51859 | 14.93424 | 15.34989 | .268642047 | -.147004626 | -.562651298 |
| Engagement | 21 | 14.52026 | 14.93591 | 15.35156 | .266969386 | -.148677287 | -.564323959 |
| Engagement | 22 | 14.52194 | 14.93758 | 15.35323 | .265296724 | -.150349948 | -.565996620 |
| Engagement | 23 | 14.52361 | 14.93926 | 15.35490 | .263624063 | -.152022609 | -.567669282 |
| Engagement | 24 | 14.52528 | 14.94093 | 15.35658 | .261951402 | -.153695270 | -.569341943 |
| Engagement | 25 | 14.52696 | 14.94260 | 15.35825 | .260278741 | -.155367931 | -.571014604 |
| Engagement | 26 | 14.52863 | 14.94427 | 15.35992 | .258606080 | -.157040593 | -.572687265 |
| Engagement | 27 | 14.53030 | 14.94595 | 15.36159 | .256933419 | -.158713254 | -.574359926 |
| Engagement | 28 | 14.53197 | 14.94762 | 15.36327 | .255260758 | -.160385915 | -.576032587 |
| Engagement | 29 | 14.53365 | 14.94929 | 15.36494 | .253588096 | -.162058576 | -.577705248 |
| Engagement | 30 | 14.53532 | 14.95097 | 15.36661 | .251915435 | -.163731237 | -.579377910 |
| Engagement | 31 | 14.53699 | 14.95264 | 15.36828 | .250242774 | -.165403898 | -.581050571 |
| Engagement | 32 | 14.53866 | 14.95431 | 15.36996 | .248570113 | -.167076559 | -.582723232 |
| Engagement | 33 | 14.54034 | 14.95598 | 15.37163 | .246897452 | -.168749221 | -.584395893 |
| Engagement | 34 | 14.54201 | 14.95766 | 15.37330 | .245224791 | -.170421882 | -.586068554 |
| Engagement | 35 | 14.54368 | 14.95933 | 15.37498 | .243552130 | -.172094543 | -.587741215 |
| Engagement | 36 | 14.54535 | 14.96100 | 15.37665 | .241879469 | -.173767204 | -.589413876 |
| Engagement | 37 | 14.54703 | 14.96267 | 15.37832 | .240206807 | -.175439865 | -.591086538 |
| Engagement | 38 | 14.54870 | 14.96435 | 15.37999 | .238534146 | -.177112526 | -.592759199 |
| Engagement | 39 | 14.55037 | 14.96602 | 15.38167 | .236861485 | -.178785187 | -.594431860 |
| Engagement | 40 | 14.55205 | 14.96769 | 15.38334 | .235188824 | -.180457848 | -.596104521 |
| Suppression | 8 | 14.49852 | 14.51940 | 14.54027 | .288713980 | .267836727 | .246959473 |
| Suppression | 9 | 14.52487 | 14.54574 | 14.56662 | .262368231 | .241490977 | .220613724 |
| Suppression | 10 | 14.55121 | 14.57209 | 14.59297 | .236022481 | .215145227 | .194267974 |
| Suppression | 11 | 14.57756 | 14.59843 | 14.61931 | .209676731 | .188799477 | .167922224 |
| Suppression | 12 | 14.60390 | 14.62478 | 14.64566 | .183330981 | .162453728 | .141576474 |
| Suppression | 13 | 14.63025 | 14.65113 | 14.67200 | .156985231 | .136107978 | .115230724 |
| Suppression | 14 | 14.65659 | 14.67747 | 14.69835 | .130639481 | .109762228 | .088884975 |
| Suppression | 15 | 14.68294 | 14.70382 | 14.72469 | .104293732 | .083416478 | .062539225 |
| Suppression | 16 | 14.70929 | 14.73016 | 14.75104 | .077947982 | .057070728 | .036193475 |
| Suppression | 17 | 14.73563 | 14.75651 | 14.77739 | .051602232 | .030724978 | .009847725 |
| Suppression | 18 | 14.76198 | 14.78285 | *14.80373 | .025256482 | .004379229 | *-.016498025 |
| Suppression | 19 | *14.78832 | *14.80920 | 14.83008 | *-.001089268 | *-.021966521 | -.042843775 |
| Suppression | 20 | 14.81467 | 14.83555 | 14.85642 | -.027435018 | -.048312271 | -.069189524 |
| Suppression | 21 | 14.84101 | 14.86189 | 14.88277 | -.053780767 | -.074658021 | -.095535274 |
| Suppression | 22 | 14.86736 | 14.88824 | 14.90912 | -.080126517 | -.101003771 | -.121881024 |
| Suppression | 23 | 14.89371 | 14.91458 | 14.93546 | -.106472267 | -.127349520 | -.148226774 |
| Suppression | 24 | 14.92005 | 14.94093 | 14.96181 | -.132818017 | -.153695270 | -.174572524 |
| Suppression | 25 | 14.94640 | 14.96728 | 14.98815 | -.159163767 | -.180041020 | -.200918274 |
| Suppression | 26 | 14.97274 | 14.99362 | 15.01450 | -.185509516 | -.206386770 | -.227264023 |
| Suppression | 27 | 14.99909 | 15.01997 | 15.04084 | -.211855266 | -.232732520 | -.253609773 |
| Suppression | 28 | 15.02544 | 15.04631 | 15.06719 | -.238201016 | -.259078270 | -.279955523 |
| Suppression | 29 | 15.05178 | 15.07266 | 15.09354 | -.264546766 | -.285424019 | -.306301273 |
| Suppression | 30 | 15.07813 | 15.09900 | 15.11988 | -.290892516 | -.311769769 | -.332647023 |
| Suppression | 31 | 15.10447 | 15.12535 | 15.14623 | -.317238266 | -.338115519 | -.358992773 |
| Suppression | 32 | 15.13082 | 15.15170 | 15.17257 | -.343584015 | -.364461269 | -.385338522 |
| Suppression | 33 | 15.15716 | 15.17804 | 15.19892 | -.369929765 | -.390807019 | -.411684272 |
| Suppression | 34 | 15.18351 | 15.20439 | 15.22526 | -.396275515 | -.417152769 | -.438030022 |
| Suppression | 35 | 15.20986 | 15.23073 | 15.25161 | -.422621265 | -.443498518 | -.464375772 |
| Suppression | 36 | 15.23620 | 15.25708 | 15.27796 | -.448967015 | -.469844268 | -.490721522 |
| Suppression | 37 | 15.26255 | 15.28342 | 15.30430 | -.475312765 | -.496190018 | -.517067271 |
| Suppression | 38 | 15.28889 | 15.30977 | 15.33065 | -.501658514 | -.522535768 | -.543413021 |
| Suppression | 39 | 15.31524 | 15.33612 | 15.35699 | -.528004264 | -.548881518 | -.569758771 |
| Suppression | 40 | 15.34158 | 15.36246 | 15.38334 | -.554350014 | -.575227267 | -.596104521 |
| Arousal control | 4 | 14.49852 | 14.90069 | 15.30285 | .288713980 | -.113451031 | -.515616042 |
| Arousal control | 5 | 14.50355 | 14.90572 | 15.30788 | .283683450 | -.118481561 | -.520646572 |
| Arousal control | 6 | 14.50858 | 14.91075 | 15.31291 | .278652921 | -.123512091 | -.525677102 |
| Arousal control | 7 | 14.51361 | 14.91578 | 15.31794 | .273622391 | -.128542621 | -.530707632 |
| Arousal control | 8 | 14.51864 | 14.92081 | 15.32297 | .268591861 | -.133573151 | -.535738162 |
| Arousal control | 9 | 14.52367 | 14.92584 | 15.32800 | .263561331 | -.138603680 | -.540768692 |
| Arousal control | 10 | 14.52870 | 14.93087 | 15.33303 | .258530801 | -.143634210 | -.545799222 |
| Arousal control | 11 | 14.53373 | 14.93590 | 15.33806 | .253500271 | -.148664740 | -.550829752 |
| Arousal control | 12 | 14.53876 | 14.94093 | 15.34309 | .248469741 | -.153695270 | -.555860281 |
| Arousal control | 13 | 14.54379 | 14.94596 | 15.34812 | .243439211 | -.158725800 | -.560890811 |
| Arousal control | 14 | 14.54883 | 14.95099 | 15.35316 | .238408681 | -.163756330 | -.565921341 |
| Arousal control | 15 | 14.55386 | 14.95602 | 15.35819 | .233378151 | -.168786860 | -.570951871 |
| Arousal control | 16 | 14.55889 | 14.96105 | 15.36322 | .228347621 | -.173817390 | -.575982401 |
| Arousal control | 17 | 14.56392 | 14.96608 | 15.36825 | .223317091 | -.178847920 | -.581012931 |
| Arousal control | 18 | 14.56895 | 14.97111 | 15.37328 | .218286561 | -.183878450 | -.586043461 |
| Arousal control | 19 | 14.57398 | 14.97614 | 15.37831 | .213256031 | -.188908980 | -.591073991 |
| Arousal control | 20 | 14.57901 | 14.98117 | 15.38334 | .208225501 | -.193939510 | -.596104521 |
| Distraction | 4 | 14.49852 | 14.91475 | 15.33097 | .288713980 | -.127513196 | -.543740373 |
| Distraction | 5 | 14.50179 | 14.91802 | 15.33425 | .285441221 | -.130785955 | -.547013132 |
| Distraction | 6 | 14.50507 | 14.92129 | 15.33752 | .282168462 | -.134058715 | -.550285891 |
| Distraction | 7 | 14.50834 | 14.92457 | 15.34079 | .278895703 | -.137331474 | -.553558650 |
| Distraction | 8 | 14.51161 | 14.92784 | 15.34407 | .275622943 | -.140604233 | -.556831410 |
| Distraction | 9 | 14.51488 | 14.93111 | 15.34734 | .272350184 | -.143876992 | -.560104169 |
| Distraction | 10 | 14.51816 | 14.93438 | 15.35061 | .269077425 | -.147149752 | -.563376928 |
| Distraction | 11 | 14.52143 | 14.93766 | 15.35388 | .265804665 | -.150422511 | -.566649688 |
| Distraction | 12 | 14.52470 | 14.94093 | 15.35716 | .262531906 | -.153695270 | -.569922447 |
| Distraction | 13 | 14.52797 | 14.94420 | 15.36043 | .259259147 | -.156968030 | -.573195206 |
| Distraction | 14 | 14.53125 | 14.94747 | 15.36370 | .255986388 | -.160240789 | -.576467965 |
| Distraction | 15 | 14.53452 | 14.95075 | 15.36697 | .252713628 | -.163513548 | -.579740725 |
| Distraction | 16 | 14.53779 | 14.95402 | 15.37025 | .249440869 | -.166786307 | -.583013484 |
| Distraction | 17 | 14.54107 | 14.95729 | 15.37352 | .246168110 | -.170059067 | -.586286243 |
| Distraction | 18 | 14.54434 | 14.96057 | 15.37679 | .242895351 | -.173331826 | -.589559002 |
| Distraction | 19 | 14.54761 | 14.96384 | 15.38007 | .239622591 | -.176604585 | -.592831762 |
| Distraction | 20 | 14.55088 | 14.96711 | 15.38334 | .236349832 | -.179877344 | -.596104521 |
| Reappraisal | 8 | 14.49852 | 15.36880 | 16.23908 | .288713980 | -.581568203 | -1.451850386 |
| Reappraisal | 9 | 14.47178 | 15.34206 | 16.21234 | .315456039 | -.554826145 | -1.425108328 |
| Reappraisal | 10 | 14.44504 | 15.31532 | 16.18560 | .342198097 | -.528084086 | -1.398366269 |
| Reappraisal | 11 | 14.41829 | 15.28858 | 16.15886 | .368940155 | -.501342028 | -1.371624211 |
| Reappraisal | 12 | 14.39155 | 15.26183 | 16.13212 | .395682214 | -.474599970 | -1.344882153 |
| Reappraisal | 13 | 14.36481 | 15.23509 | 16.10537 | .422424272 | -.447857911 | -1.318140095 |
| Reappraisal | 14 | 14.33807 | 15.20835 | 16.07863 | .449166330 | -.421115853 | -1.291398036 |
| Reappraisal | 15 | 14.31133 | 15.18161 | 16.05189 | .475908388 | -.394373795 | -1.264655978 |
| Reappraisal | 16 | 14.28458 | 15.15487 | 16.02515 | .502650447 | -.367631737 | -1.237913920 |
| Reappraisal | 17 | 14.25784 | 15.12812 | 15.99841 | .529392505 | -.340889678 | -1.211171861 |
| Reappraisal | 18 | 14.23110 | 15.10138 | 15.97166 | .556134563 | -.314147620 | -1.184429803 |
| Reappraisal | 19 | 14.20436 | 15.07464 | 15.94492 | .582876622 | -.287405562 | -1.157687745 |
| Reappraisal | 20 | 14.17762 | 15.04790 | 15.91818 | .609618680 | -.260663503 | -1.130945687 |
| Reappraisal | 21 | 14.15087 | 15.02116 | 15.89144 | .636360738 | -.233921445 | -1.104203628 |
| Reappraisal | 22 | 14.12413 | 14.99441 | 15.86470 | .663102796 | -.233921445 | -1.077461570 |
| Reappraisal | 23 | 14.09739 | 14.96767 | 15.83795 | .689844855 | -.180437329 | -1.050719512 |
| Reappraisal | 24 | 14.07065 | 14.94093 | 15.81121 | .716586913 | -.153695270 | -1.023977453 |
| Reappraisal | 25 | 14.04391 | 14.91419 | 15.78447 | .743328971 | -.126953212 | -.997235395 |
| Reappraisal | 26 | 14.01716 | 14.88745 | 15.75773 | .770071029 | -.100211154 | -.970493337 |
| Reappraisal | 27 | 13.99042 | 14.86070 | 15.73099 | .796813088 | -.073469095 | -.943751279 |
| Reappraisal | 28 | 13.96368 | 14.83396 | 15.70424 | .823555146 | -.046727037 | -.917009220 |
| Reappraisal | 29 | 13.93694 | 14.80722 | 15.67750 | .850297204 | -.019984979 | -.890267162 |
| Reappraisal | 30 | 13.91019 | *14.78048 | 15.65076 | .877039263 | *.006757079 | -.863525104 |
| Reappraisal | 31 | 13.88345 | 14.75373 | 15.62402 | .903781321 | .033499138 | -.836783045 |
| Reappraisal | 32 | 13.85671 | 14.72699 | 15.59728 | .930523379 | .060241196 | -.810040987 |
| Reappraisal | 33 | 13.82997 | 14.70025 | 15.57053 | .957265437 | .086983254 | -.783298929 |
| Reappraisal | 34 | 13.80323 | 14.67351 | 15.54379 | .984007496 | .113725313 | -.756556871 |
| Reappraisal | 35 | 13.77648 | 14.64677 | 15.51705 | 1.010749554 | .140467371 | -.729814812 |
| Reappraisal | 36 | 13.74974 | 14.62002 | 15.49031 | 1.037491612 | .167209429 | -.703072754 |
| Reappraisal | 37 | 13.72300 | 14.59328 | 15.46356 | 1.064233671 | .193951487 | -.676330696 |
| Reappraisal | 38 | 13.69626 | 14.56654 | 15.43682 | 1.090975729 | .220693546 | -.649588638 |
| Reappraisal | 39 | 13.66952 | 14.53980 | 15.41008 | 1.117717787 | .247435604 | -.622846579 |
| Reappraisal | 40 | 13.64277 | 14.51306 | 15.38334 | 1.144459845 | .274177662 | -.596104521 |

Note. ER = emotional regulation; ESA = expected symptom activation.

*cut-off value to generate a greater symptoms activation.

**3. Graphical representation of the Exploratory Analysis of RESS**

**Figure 1**

*Graphical representation of the Exploratory Graph Analysis of the RESS*


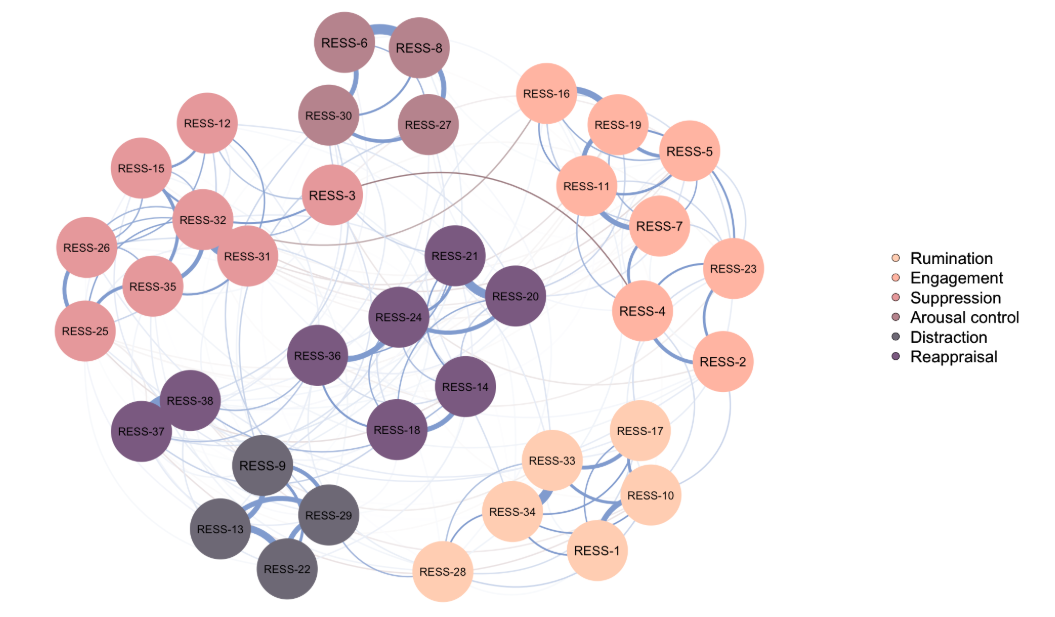


*Note*. Graphical representation of the Exploratory Graph Analysis of the RESS*.* Each node represents the RESS items.

The color of the nodes represents the community to each item is most assigned to. Blue edges represent the positive connections between nodes and the brown edges represent the negative connections. The thickness of the edges represents the magnitude of its connections.

**4. Strength centrality plot of RESS items**

**Figure 2**

*Strength centrality plot of RESS items*


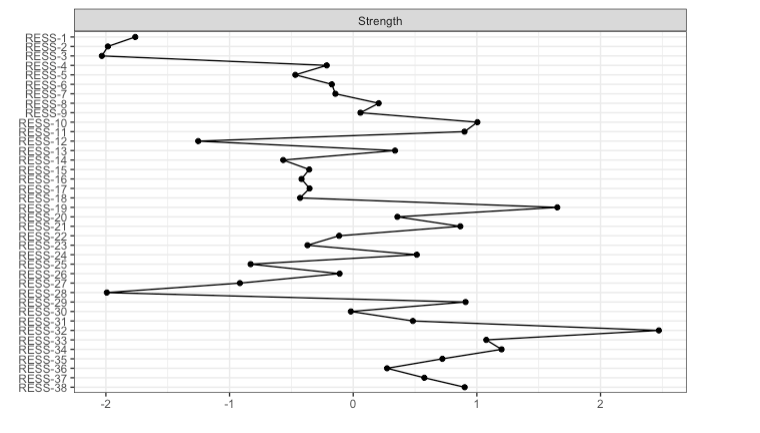


*Note.* Items strength values of RESS.

The vertices 32 and 19 are the strongest in the network of RESS. On the other hand, the vertices 28, 3, and 2 are the least strong in the network.

1. **Stability Plots Mixed Graphical Model**

**Figure 3**

*Edge-weight Accuracy*

A)


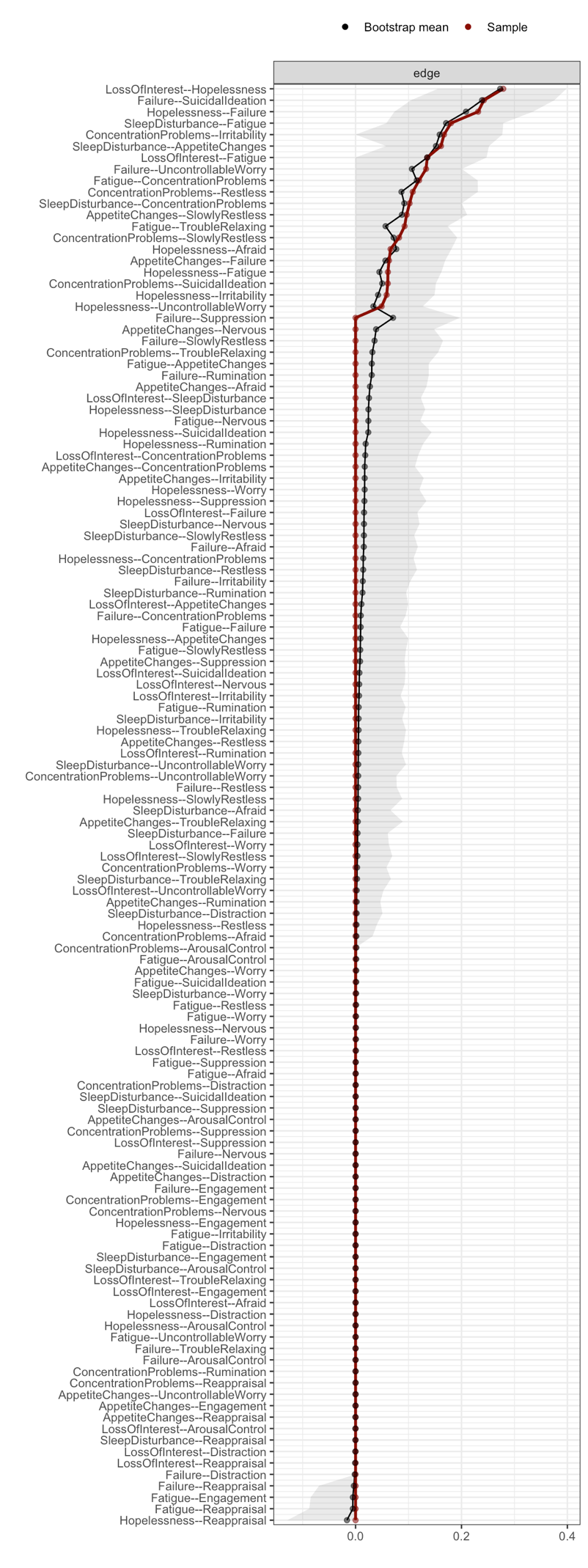


B)


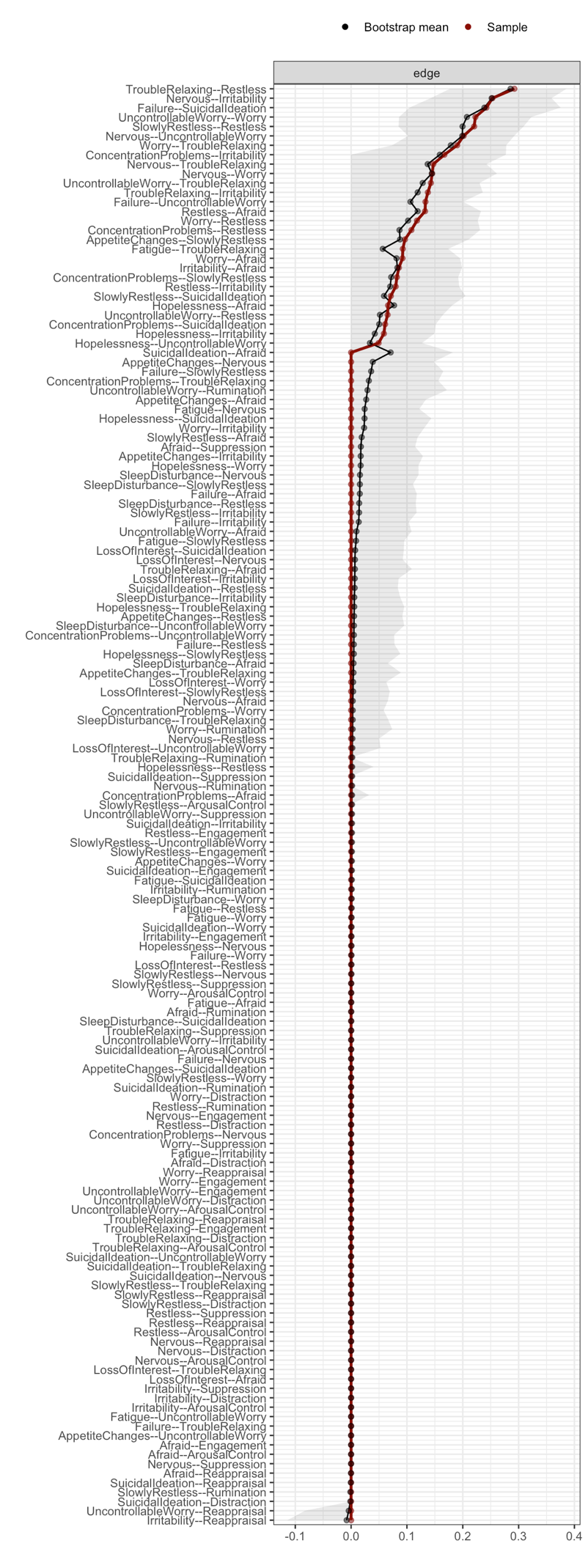


C)


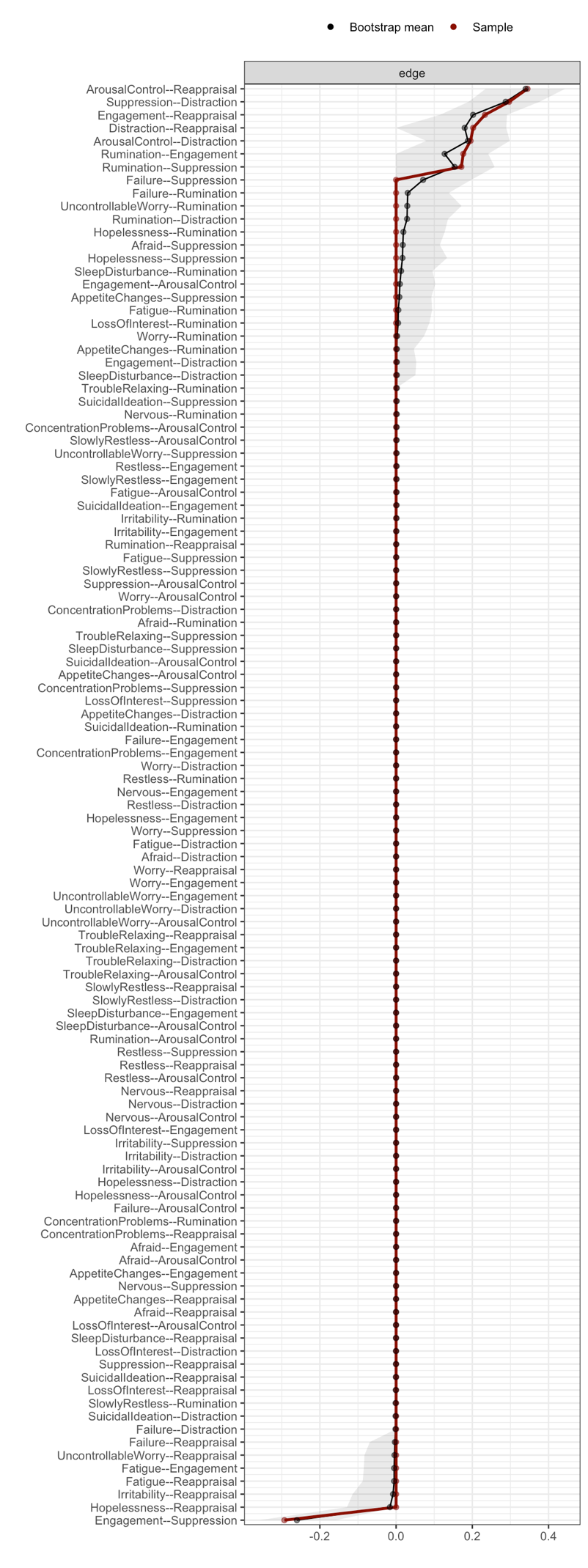


*Note.* Bootstrap confidence intervals were computed for the estimated edge-weights within the network. The red line indicates the sample values, while the gray area represents the bootstrap confidence intervals. Each horizontal line corresponds to an edge within the network, arranged from the highest to the lowest edge-weight.

**Figure 4**

*Centrality Stability*


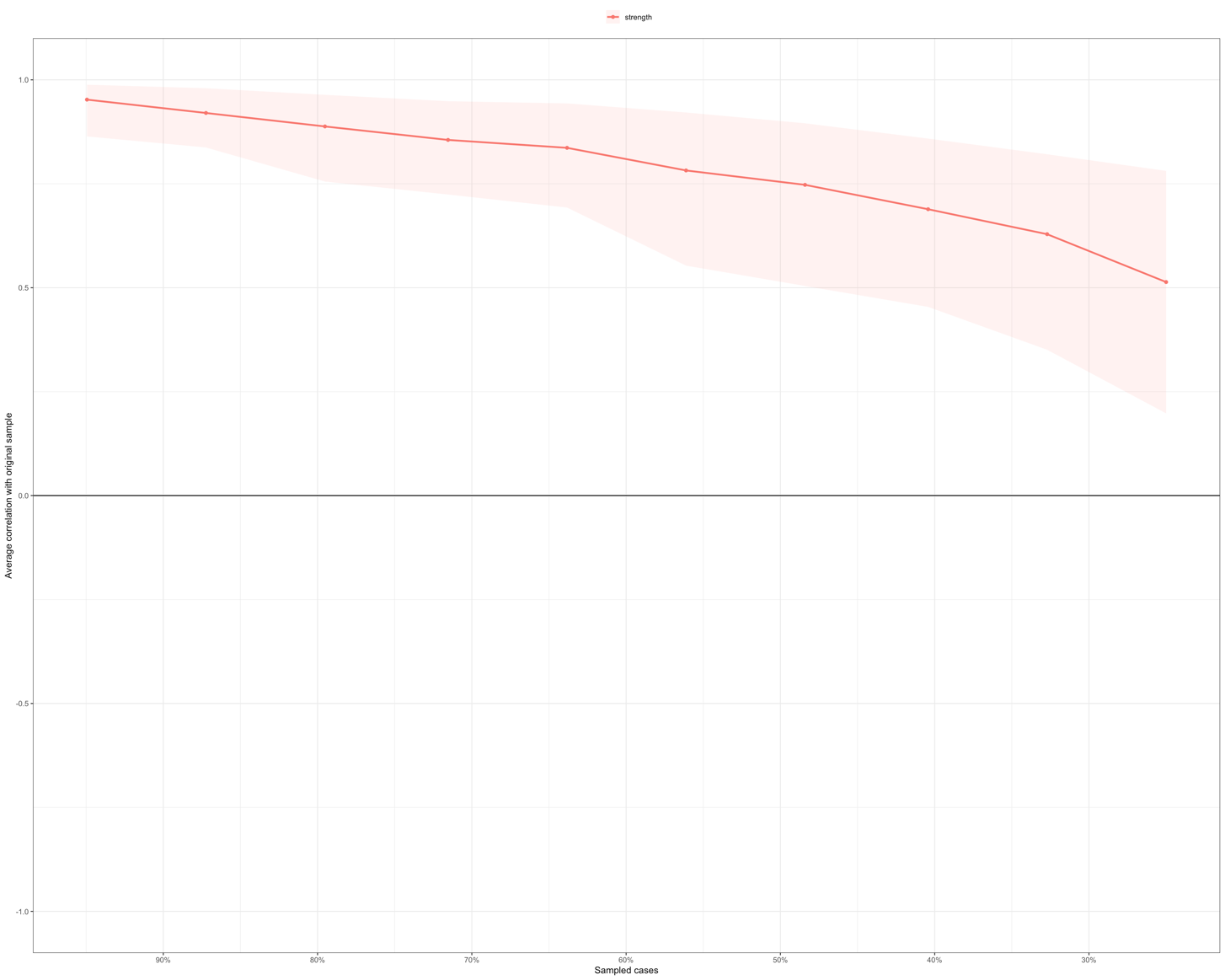


*Note.* The average correlations among centrality indices of networks obtained through sampling with individuals excluded were calculated in comparison to the original sample. The line illustrate the average, while the shaded areas indicate the range from the 2.5th to the 97.5th quantile.

**Figure 5**

*Bootstrapped Difference Tests*


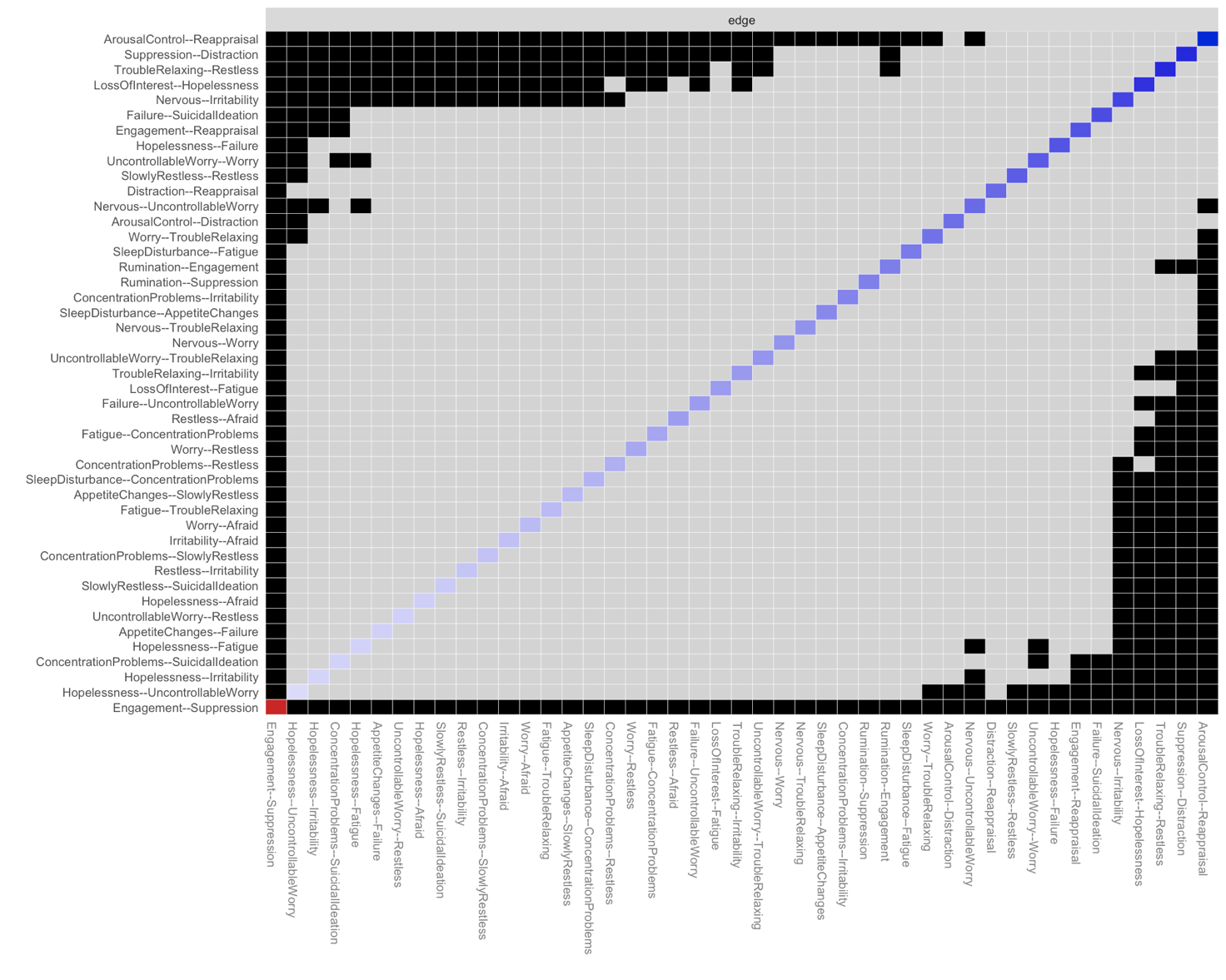


B)


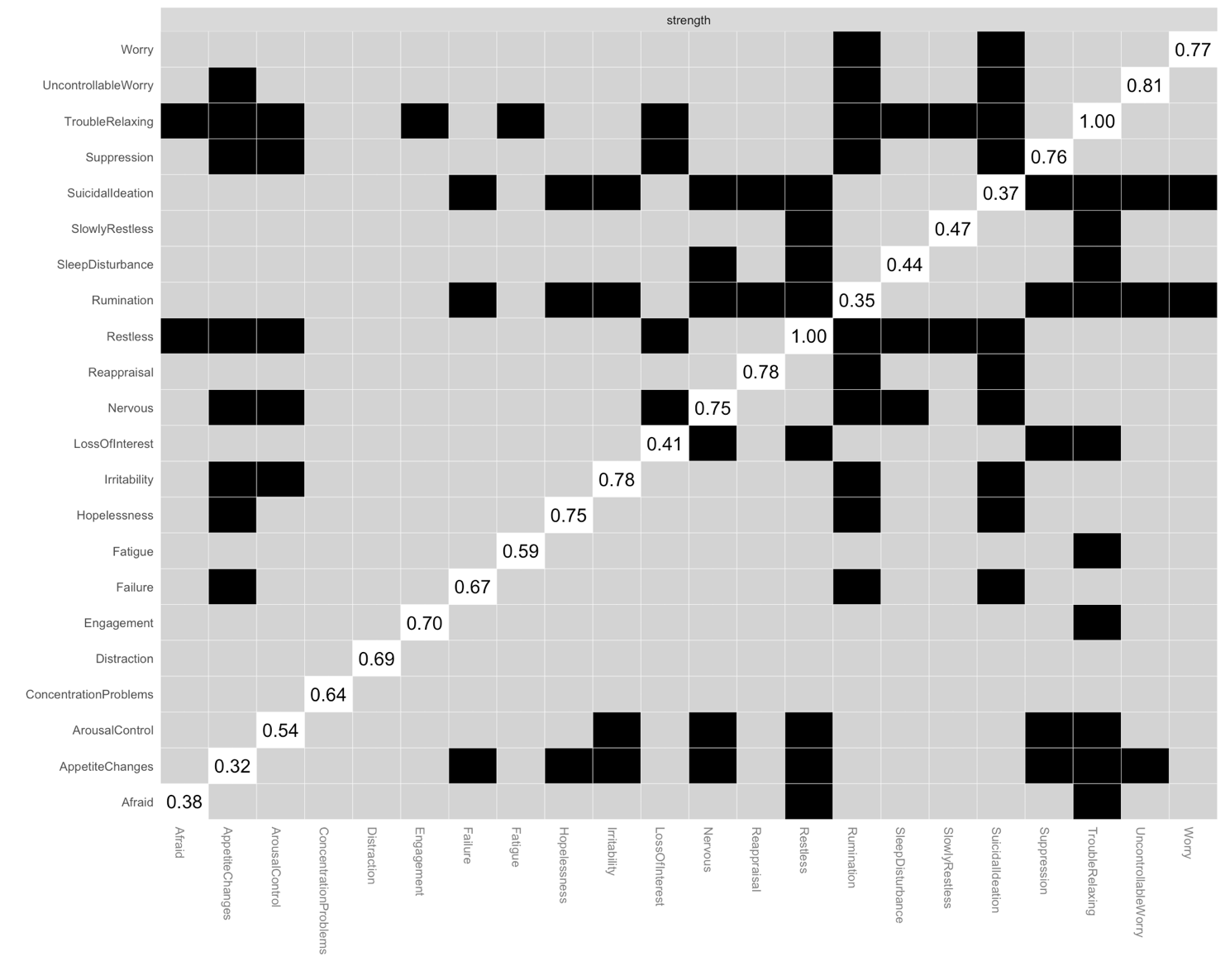


*Note.* Bootstrapped difference tests (with a significance level of α = 0.05) were conducted between non-zero edge weights within the estimated relative importance network (above) and the node in-strength of the 22 network nodes (below). Gray-boxed nodes or edges indicate instances where no significant differences were detected, while black-boxed ones highlights significant differences.

1. **Analysis of the confounding effects of sociodemographic variables**

The potentially confounding effects of sociodemographic variables was tested by including these variables in the estimation of the network of interactions between emotion regulation strategies and symptoms of depression and anxiety (Figure 6). Following the procedures proposed by van Borkulo et al. (2015), we also regressed each variable representing emotion regulation strategies and the symptoms of depression and anxiety on the sociodemographic variables and used residuals to depict the network (Figure 7).

**Figure 6**

*Network of interactions between sociodemographic variables, emotion regulation strategies and symptoms of depression and anxiety*


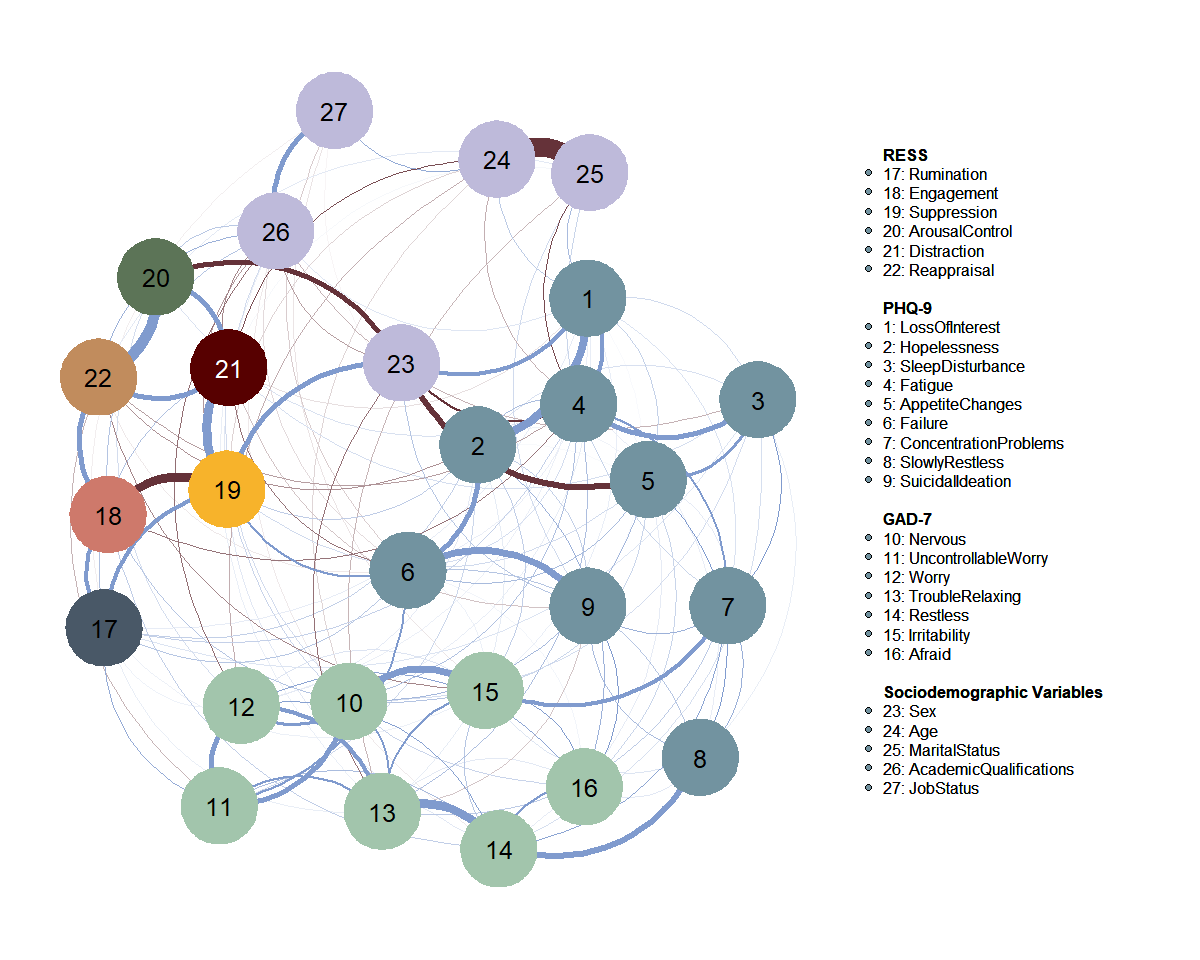


The network visualizes the interactions between sociodemographic variables, emotion regulation strategies, and symptoms of depression and anxiety. In this network, each variable is represented by a colored circle, or node. The blue nodes (1 to 9) correspond to depression symptoms measured by the PHQ-9, while the green nodes (10 to 16) correspond to anxiety symptoms measured by the GAD-7. Sociodemographic variables are represented by purple nodes (23 to 27). Emotion regulation strategies are depicted by yellow, orange, pink, dark green, dark red, and dark blue nodes (17 to 22). The interactions between these variables are illustrated by lines, or edges, where the thickness of the edges signifies the strength of the interaction between two nodes (thicker lines indicate stronger correlations). The color of the edges denotes the direction and type of interaction, with blue indicating positive correlations and red indicating negative correlations. Additionally, it is important to note that in this network, the variables related to sex (node 23) and age (node 24) cannot be influenced by other variables, which is a limitation.

**Figure 7**

*Strength centrality plot of network of interactions between sociodemographic variables, emotion regulation strategies and symptoms of depression and anxiety*


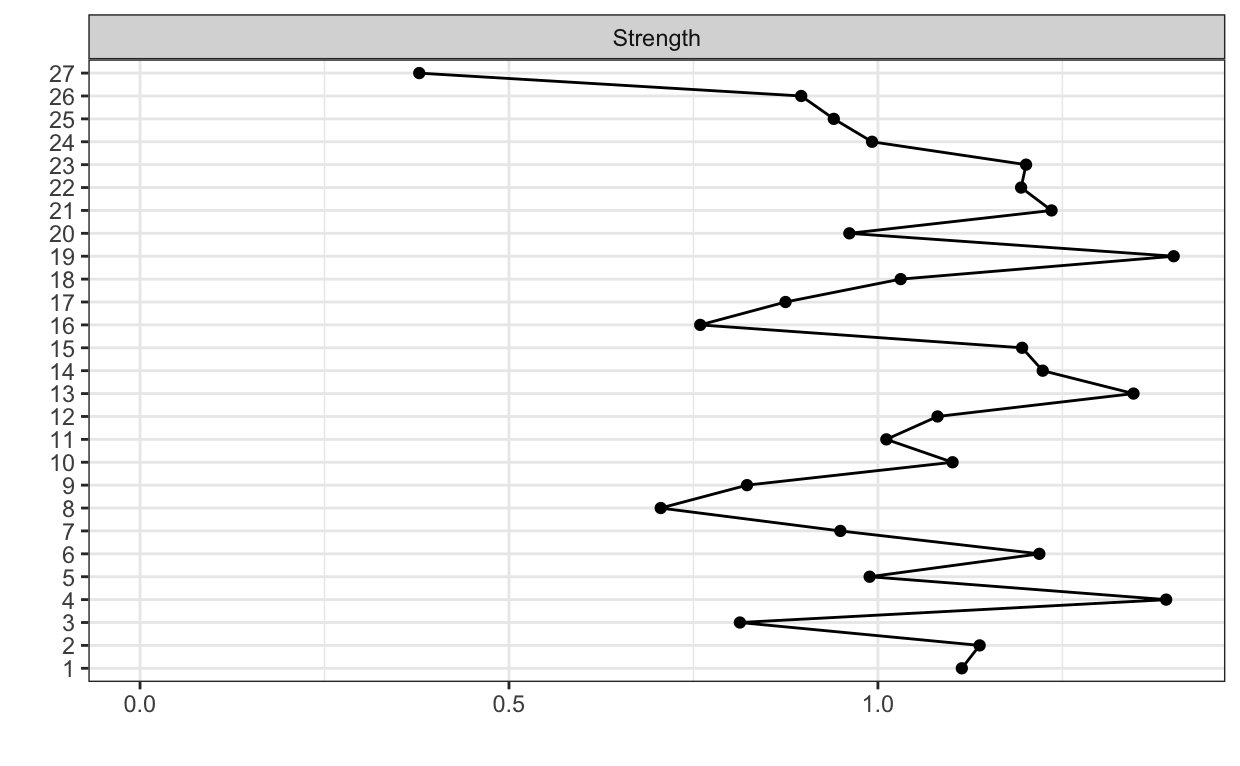


Figure 7 shows the items strength values of sociodemographic variables, emotion regulation strategies, and symptoms of depression and anxiety. The vertices are as follows: 1 – loss of interest; 2 – hopelessness; 3 – sleep disturbance; 4 – fatigue; 5 – appetite changes; 6 – failure; 7 – concentration problems; 8 – slowly/restless; 9 – suicidal ideation; 10 – nervous; 11 – uncontrollable worry; 12 – worry; 13 – trouble relaxing; 14 – restless; 15 – irritability; 16 – afraid; 17 – rumination; 18 – engagement; 19 – suppression; 20 – arousal control; 21 – distraction; 22 – reappraisal; 23 – sex; 24 – age; 25 – marital status; 26 – academic qualifications and 27 – job status. Vertices 19 (suppression), 3 (sleep disturbance), and 13 (trouble relaxing) are the strongest in the network. On the other hand, the vertices 27 (job status), 8 (slowly/restless), and 16 (afraid) are the least strong in the network.

**Figure 8**

*Network of interactions between emotion regulation strategies and symptoms of depression and anxiety after partialling out sociodemographic variables*

*
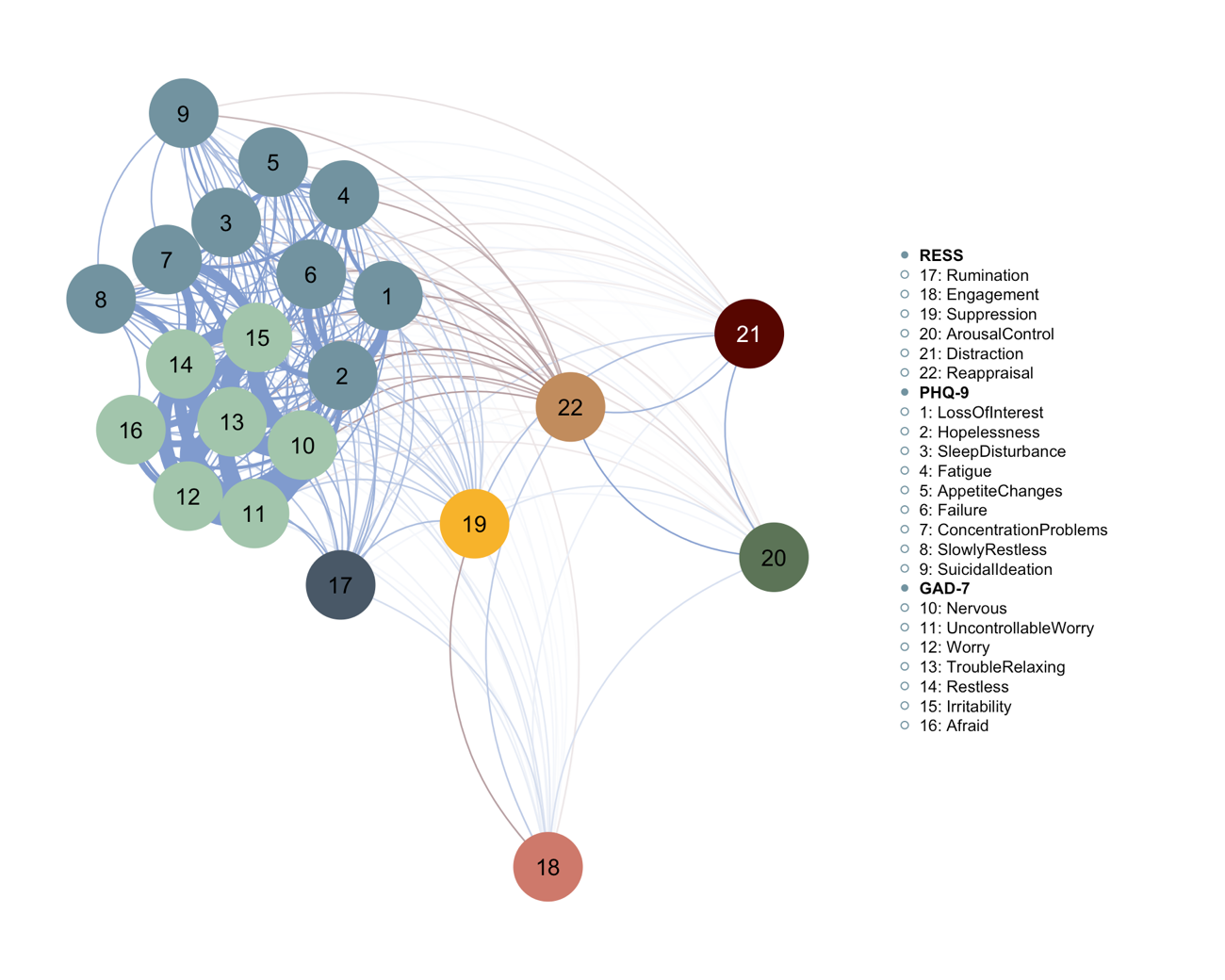
*

Network depicts the interactions between emotional regulation strategies (represented by the colors yellow, orange, pink, dark green, dark red and dark blue), depression (represented by the color blue) and anxiety (represented by the color green) after partialling out sociodemographic variables. The connections between these variables, depicted as lines - edges, vary in thickness to indicate the strength of the interactions, with thicker lines signifying stronger relationships. The color of the edges conveys the direction of the interaction: red edges denote negative interactions, while blue edges denote positive interactions.

**Figure 9**

*Strength centrality plot of network of interactions between emotion regulation strategies and symptoms of depression and anxiety*


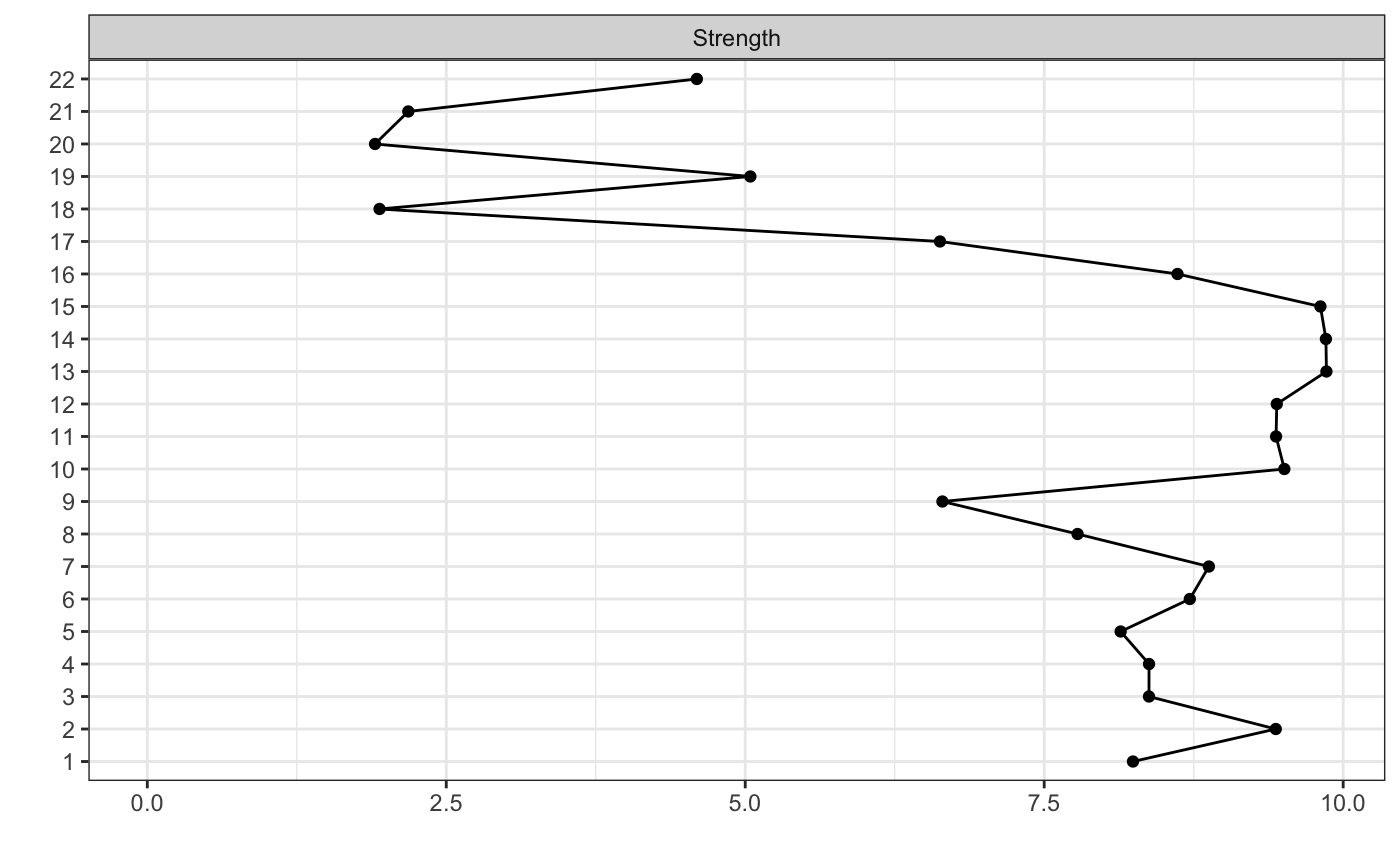


Figure 9 shows the centrality strength of network vertices after partialling out SD variables. Vertices are ranked based on their strength within the network. The vertices are as follows: 1 – loss of interest; 2 – hopelessness; 3 – sleep disturbance; 4 – fatigue; 5 – appetite changes; 6 – failure; 7 – concentration problems; 8 – slowly/restless; 9 – suicidal ideation; 10 – nervous; 11 – uncontrollable worry; 12 – worry; 13 – trouble relaxing; 14 – restless; 15 – irritability; 16 – afraid; 17 – rumination; 18 – engagement; 19 – suppression; 20 – arousal control; 21 – distraction; 22 – reappraisal. Vertices 13 (trouble relaxing), 14 (restless), and 15 (irritability) exhibit the highest strength, indicating they are the most influential nodes in the network. In contrast, vertices 18 (engagement), 20 (arousal control), and 21 (distraction) show the lowest strength, indicating they are the least influential nodes.
